# Efficacy and Mechanism of Traditional Medicinal Plants and Bioactive Compounds against Clinically Important Pathogens

**DOI:** 10.3390/antibiotics8040257

**Published:** 2019-12-09

**Authors:** Suresh Mickymaray

**Affiliations:** Department of Biology, College of Science, Al-Zulfi-, Majmaah University, Majmaah 11952, Saudi Arabia; s.maray@mu.edu.sa

**Keywords:** traditional medicinal plants, bioactive compounds, antimicrobial activities, mechanisms

## Abstract

Traditional medicinal plants have been cultivated to treat various human illnesses and avert numerous infectious diseases. They display an extensive range of beneficial pharmacological and health effects for humans. These plants generally synthesize a diverse range of bioactive compounds which have been established to be potent antimicrobial agents against a wide range of pathogenic organisms. Various research studies have demonstrated the antimicrobial activity of traditional plants scientifically or experimentally measured with reports on pathogenic microorganisms resistant to antimicrobials. The antimicrobial activity of medicinal plants or their bioactive compounds arising from several functional activities may be capable of inhibiting virulence factors as well as targeting microbial cells. Some bioactive compounds derived from traditional plants manifest the ability to reverse antibiotic resistance and improve synergetic action with current antibiotic agents. Therefore, the advancement of bioactive-based pharmacological agents can be an auspicious method for treating antibiotic-resistant infections. This review considers the functional and molecular roles of medicinal plants and their bioactive compounds, focusing typically on their antimicrobial activities against clinically important pathogens.

## 1. Introduction

The incidence of microbial infectious diseases and their hitches consistently elevates, mostly due to microbial drug resistance to presently offered antimicrobial agents [1]. These multidrug-resistant microbes cause various infections globally and are connected with greater levels of morbidity and mortality [2]. These augmentations of antibiotic resistance and higher recurrence rates of such common infections have a great impact on our society [3,4,5]. Several investigations associated with antimicrobial resistance predict that the mortality toll owing to antimicrobial resistance may exceed 10 million by 2050, theoretically leading to greater mortality in the context of other infectious diseases and malignancies [6]. It is well known that infections are generally difficult to treat due to the development of biofilm in the host, which aids the proliferation of microbes as well as the aggressiveness of the infections [7]. Studies have also well-established that the physical structures of biofilm establishing organisms confer natural resistance to hostile environments, including antimicrobial agents [8]. Therefore, it is an urgent requirement to generate novel antimicrobial drugs which can inhibit the development of, or abolish the complete biofilms, and hence increase the vulnerability of microbes to antimicrobials. The requisite for new antimicrobials which could meritoriously fight against antimicrobial resistant clinical pathogens is extremely augmented.

Plant-derived antimicrobials have been established to be one of the most auspicious sources considered as safe due to their natural origin when compared with synthetic compounds [9,10]. There is an accumulating interest in the practice of either crude extract of medicinal plants, as well as the screening plant-derived compounds as an alternative therapy for microbial infections [11]. Plants generally produce a diverse range of bioactive compounds which have been widely used in clinical practice [12]. Remarkably, a significant number of marketed drugs are obtained from nature or result in natural products through either chemical transformations or de novo synthesis [13]. Plant-derived compounds are a group of secondary metabolites that are used to treat chronic as well as infectious diseases. These traditional medicinal plants or active compounds remain included as part of the habitual treatment of various maladies [9]. These compounds could have other target sites than conventional antimicrobials as well as diverse mechanisms of action against pathogenic microbes. An electronic search was performed using PubMed, Science Direct, and Google Scholar using the keywords “medicinal plants” AND “bioactive compounds” AND “antimicrobial activities” AND “antibiotic resistance” in “Title/Abstract/Keywords” without date restriction in order to identify all published studies (in vitro, in vivo, clinical and case-control) that have investigated the connection between medicinal plants and their antimicrobial effects. Antimicrobial mechanisms were gathered and for review.

## 2. Traditional Medicinal Plants

The species of the plant kingdom are estimated to number about 500,000 and only a minor portion of them have been investigated for antimicrobial activity [9,14]. Traditional medicinal plants can be cultivated by humans over centuries without existing systematic standards and analysis due to their safety and efficacy. Hence, bioactive compounds derived from these medicinal plants apparently have more potential to succeed in toxicology screening when compared with the de novo synthesis of chemicals. The cumulative attention on traditional ethnomedicine may lead to the revealing of innovative therapeutic agents since traditional medicinal plant contains potential antimicrobial components that are beneficial for the development of pharmaceutical agents for the therapy of ailments. Nowadays, studies are progressively turning their consideration to traditional medicine and advancing better drugs to treat diabetes, cancer, and microbial infections [15,16]. A large number of studies have been piloted using medicinal plant extracts and their active principles on bacteria, fungi, algae, and viruses in different localities of the world [9,10]. Various families of traditional medicinal plants have been scientifically tested for their antimicrobial activities and are presented in Table 1. The extracts of plant organs, namely the root, stem, rhizome, bulb, leaf, bark, flower, fruit, and seed, may encompass distinctive phytochemicals with antimicrobial activities [17]. It is well-known that sole plant species of traditional medicine are habitually used to heal a great number of infections or diseases [18]. The plant extracts with an antiquity of folk use should be confirmed using contemporary methods for activities against human pathogens with the intention of identifying potential novel therapeutic drugs.

### Phytocomponent Fractions and Antimicrobial Methods

Fresh or dried plant extracts were prepared using aqueous and different organic solvents in traditional extraction techniques (maceration, percolation, Soxhlet extraction). During the extraction method, the solvents penetrate into the plant material and dissolve active compounds with a related polarity [62]. At the completion of the technique, solvents have been vaporized, resulting in the formation of a concentrated mixture that yields the active compounds [63]. A successful extraction is mainly reliant on the nature of the solvent utilized during the extraction. The most regularly established extracts are aqueous extract followed by organic solvents, which include using methanol, ethanol, hexane, isopropanol, ethyl acetate, benzene, acetone, chloroform, and dichloromethane [64].

Two popular types of antibacterial susceptibility test, namely diffusion and dilution methods, are generally performed to determine the antibacterial efficacy of the plant materials. The method of diffusion is a screening test to classify bacteria that aid susceptibility or resistance to the tested plant material based on the size or diameter of the inhibition zone [62]. On the other hand, the activity of plant materials is determined as minimum inhibitory concentration (MIC) in the dilution method. In the MIC method, the lowest concentration is capable of inhibiting bacterial growth. Redox indicators and turbidity are most often measured for the analysis of results in broth dilution methods. The turbidity can be calculated colorimetrically while changing the indicator color represents the inhibition of bacterial growth [62]. The screening of traditional plant extracts has been of great attention to researchers investigating novel bioactive compounds effective in the treatment of microbial infections. Plant extracts exhibit: (a) direct antimicrobial activity presenting effects on metabolism and development of microbes and (b) indirect activity as antibiotic resistance adapting substances which, joint with antibiotics, upsurge their efficiency. Numerous studies have considered the antimicrobial screening of traditional plant extracts. The studies of medicinal plants from diverse topographical areas include: Armenia [65], Iran [66], Mexico [67], Saudi Arabia [68], Libya [26], Ethiopia [64], India [63], Poland [69], Cameroon [70], Nigeria [71], and other Middle Eastern countries [72]. Based on the available information, the traditional plant extracts showed antimicrobial activity against a huge number of pathogenic bacteria, fungi, viruses, algae, protozoan, and Trypanosoma [26,63,64,66].

## 3. Bioactive Compounds (Bioactive Phytocomponents)

Traditional medicinal plants possess various chemical substances that support certain physiological and biochemical activities in the human body and they are known as phytochemicals or phytocomponents. These chemicals are non-nutritive substances used to heal various infectious diseases, as well as provide disease preventive properties [9,10]. With advances in phytochemical practices, numerous active principles have been isolated from medicinal plants and presented as a valuable drug in contemporary systems of medicine. Mostly, the pharmacological activity of medicinal plants resides in their secondary metabolites, which are relatively smaller in quantity in contrast to the primary molecules such as carbohydrates, proteins, and lipids. Plant secondary metabolites are commonly accountable for their antimicrobial properties [62]. These metabolites offer clues to manufacture new structural types of antimicrobial and antifungal chemicals that are comparatively safe to humans [62]. The classes of secondary metabolites that have greater antimicrobial properties are flavonoids (flavones, flavonols, flavanols, isoflavones, anthocyanidins), phenolic acids (hydroxybenzoic, hydroxycinnamic acids), stilbenes, lignans, quinones, tannins, coumarins (simple coumarins, furanocoumarins, pyranocoumarins), terpenoids (sesquiterpene lactones, diterpenes, triterpenes, polyterpenes), alkaloids, glycosides, saponins, lectins, steroids, and polypeptides [6,16,56,62,73,74,75,76,77,78,79,80,81,82,83]. These compounds have copious mechanisms that underlie antimicrobial activity, e.g., disturbing microbial membranes, weakening cellular metabolism, control biofilm formation, inhibiting bacterial capsule production, attenuating bacterial virulence by controlling quorum-sensing, and reducing microbial toxin production [3,4,5,6,73,74,75,76,77,78,79,80,81,82,83,84,85]. Various bioactive compounds have been scientifically tested for their antimicrobial activities and are presented in Table 2.

## 4. Mechanism of Actions of Antibacterial Bioactive Compounds

**Figure 1:**
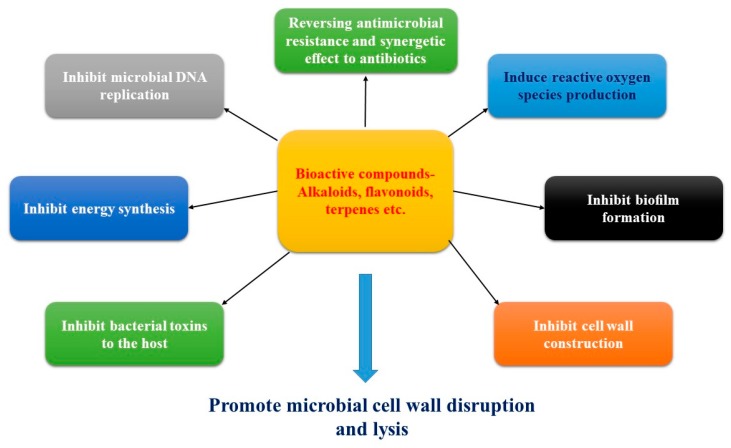
Mechanisms of antimicrobial activity of bioactive compounds.

As proven by in vitro experiments, medicinal plants produce a boundless quantity of secondary metabolites that have great antimicrobial activity [9,10,18]. These plant-produced low molecular weight antibiotics are classified according to two types, namely phytoanticipins, which are involved in microbial inhibitory actions, and phytoalexins, which are generally anti-oxidative and synthesized de novo by plants in response to microbial infection [16,74]. Plant antimicrobial secondary metabolites are generally categorized into three broad classes, namely phenolic compounds, terpenes, and alkaloids. Numerous studies have shown that the antimicrobial activity of the plant extracts and their active compounds have the following potential: to promote cell wall disruption and lysis, induce reactive oxygen species production, inhibit biofilm formation, inhibit cell wall construction, inhibit microbial DNA replication, inhibit energy synthesis, and inhibit bacterial toxins to the host [75,85,105,106,107,108,109]. In addition, these compounds may prevent antibacterial resistance as well as synergetics to antibiotics, which can ultimately kill pathogenic organisms (Figure 1).

### 4.1. Promote Cell Wall Disruption and Lysis

**Figure 2:**
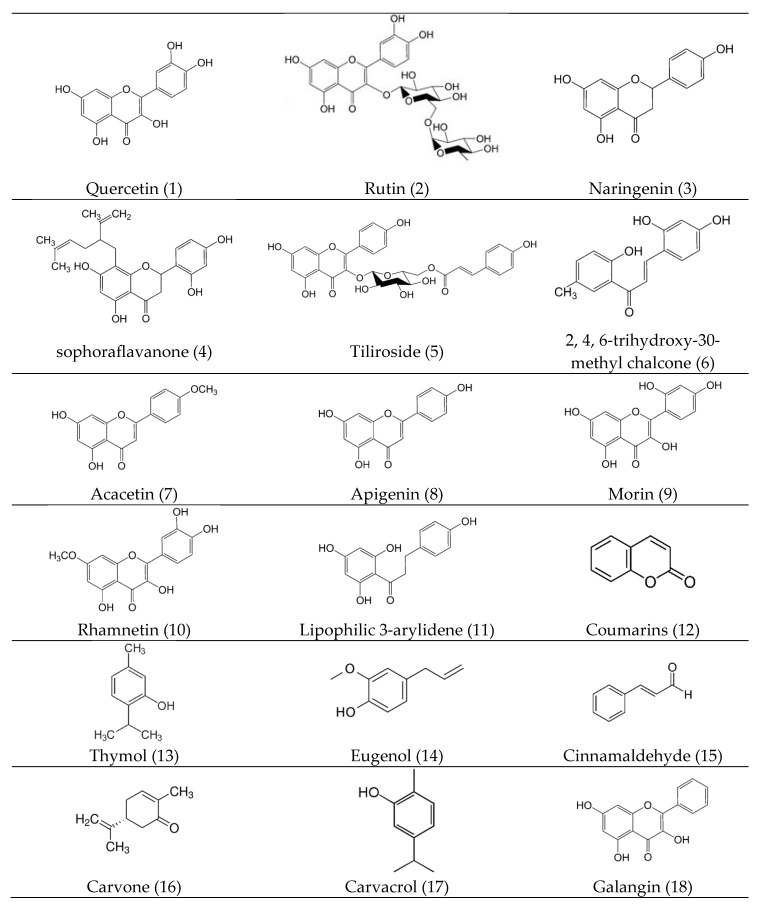

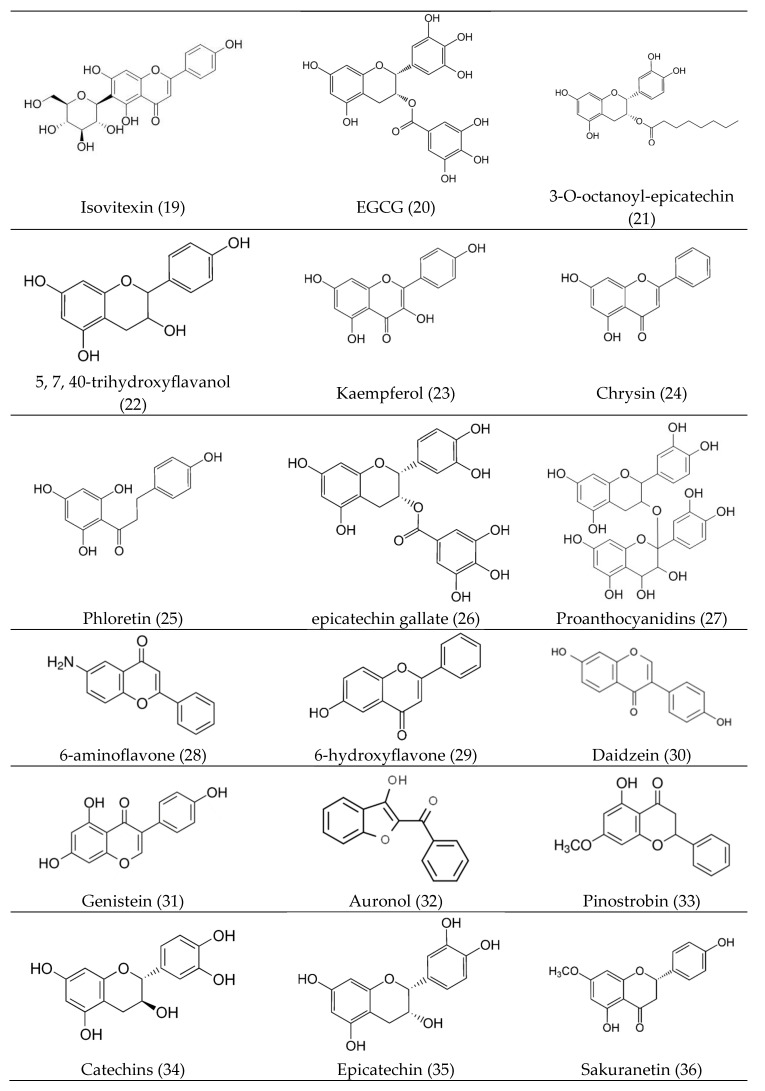

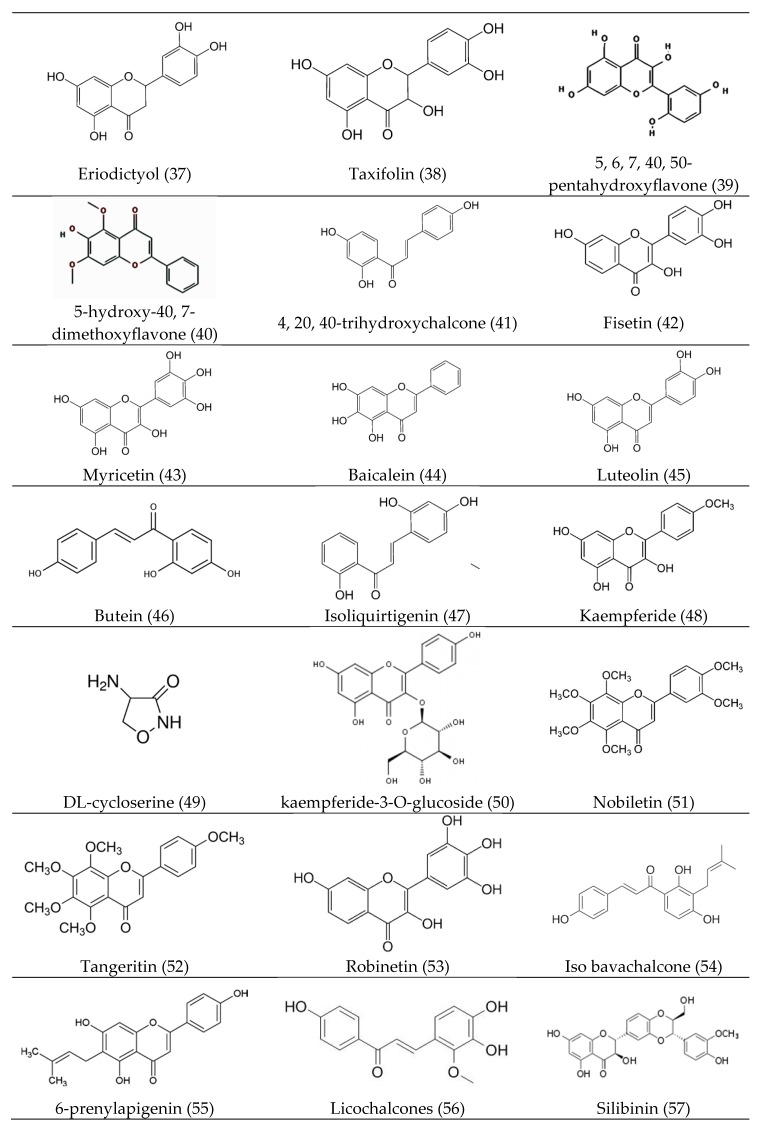

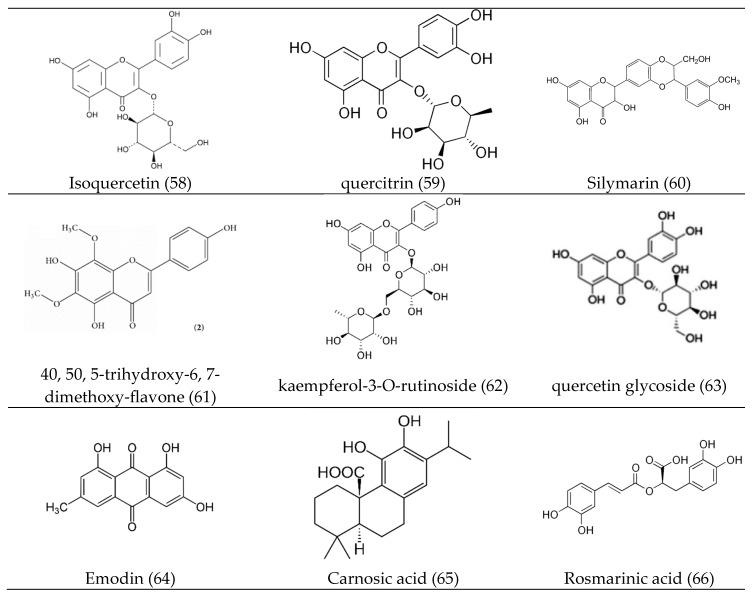
Chemical structures of antibacterial bioactive compounds.

Phenolic compounds are a family of aromatic rings consisting of a hydroxyl functional group (-OH) which is alleged to absolute toxicity to microorganisms, although increased reactions of hydroxylation result in microbial cell lysis [110]. Quinones also have aromatic rings with two ketone molecules, which enables the production of an irreversible complex with nucleophilic amino acids, resulting in greater antimicrobial properties. These potential aromatic compounds are usually targeted to microbial cell surface adhesins, membrane-bound polypeptides, enzymes, and eventually lysis of the microbes [111]. Flavonoids are hydroxylated phenolic substances which are also able to complex with bacterial cell walls and disrupt microbial membranes [75,105]. Highly active flavonoids, quercetin (1), rutin (2), naringenin (3), sophoraflavanone (4), tiliroside (5) and 2, 4, 6-trihydroxy-30-methyl chalcone (6) (Figure 2) decreased lipid bilayer thickness and fluidity levels and increased membrane permeability, supporting the leaking of intracellular protein and ions in *S. aureus* and *S. mutans* [112,113]. These compounds contribute to the synergistic effect with ampicillin and tetracycline [114]. The other active flavonoids, acacetin (7), apigenin (8), morin (9), and rhamnetin (10) (Figure 2) cause weakening of the bacterial cell wall by disarrangement and disorientation of the lipid bilayer and ultimately persuade vesicle leakage [115,116,117]. The synthetic flavonoid lipophilic 3-arylidene (11) was found to be very active against *S. aureus, S. epidermidis*, and *E. faecalis* due to a bacterial cell clump that influences the integrity of the cell wall as a result of biofilm disruption [118]. Tannins are classes of another polymeric phenolic substance, characterized as astringency, which is capable to deactivate microbial adhesins, enzymes, and membrane transporter systems [105,119]. Coumarins (12) are benzo-α-pyrones known to stimulate macrophages, which could have an adverse effect on infections [7,120]. Terpenes are organic compounds containing isoprene subunits, which involve microbial membrane disruption [121,122]. Thymol (13), eugenol (14), Cinnamaldehyde (15), carvone (16), and carvacrol (17) (Figure 2) disintegrate the external membrane of various Gram-negative bacteria, releasing LPS and increasing the permeability [123,124,125].

### 4.2. Inhibition of Biofilm Formation

The key features of bacteria developing biofilms are generally 100–1000 times more resistant to antimicrobial drugs while related to their usual planktonic forms [64]. Interestingly, numerous researchers have described how flavonoids cause the aggregation of multicellular composites of bacteria and inhibit bacterial growth after aggregation, which indicates that flavonoids are potent antibiofilm compounds. The bioactive flavonoids such as galangin (18), isovitexin (19), EGCG (20) and 3-O-octanoyl-epicatechin (21), as well as 5, 7, and 40-trihydroxyflavanol (22) induce pseudo multicellular aggregation of *S. aureus* and *S. mutans* [106,107,108,109]. Quorum sensing involves cell signaling molecules called autoinducers present in *E. coli, Vibrio cholerae*, and *S. typhi*, which is a notable regulatory factor for biofilm formation [126]. Interestingly, apigenin (8), kaempferol (23), quercetin (1), and naringenin (3) are effective antagonists of cell–cell signaling [126,127] that have been revealed to inhibit enteroaggregative biofilm formation in *E. coli* and *P. aeruginosa* in a concentration-dependent manner [128,129]. Moreover, chrysin (24), phloretin (25), naringenin (3), kaempferol (23), epicatechin gallate (26), proanthocyanidins (27), and EGCG (20) (Figure 2) inhibited N-acyl homoserine lactones-mediated QS [130,131,132]. Hydrophilic flavonoids such as 6-aminoflavone (28), 6-hydroxyflavone (29), apigenin (8), chrysin (24), daidzein (30), genistein (31), auronol (32), and phloretin (25) (Figure 2) have inhibitory effects on *E. coli* biofilm formation [133,134]. In addition, Phloretin (25) inhibited fimbriae formation in *E. coli* by reducing the expression of the curli genes (csgA, csgB) and toxin genes (hemolysin E, Shiga toxin 2) [6], eventually inhibiting the formation of biofilm. Hence, phloretin (25) is well known as an antibiotic resistant compound. Pinostrobin (33), EGCG (20) and prenylated flavonoids enhanced membrane permeability in *E. faecalis, S. aureus, E. coli*, and *P. aeruginosa, Porphyromonas gingivalis*, which is consistent with its effect on efflux-pump inhibitors and anti-biofilm formation [34,135,136].

### 4.3. Inhibition of Cell Wall Construction

The bacterial cell wall is accountable for osmoregulation, respiration, the transport mechanism, and biosynthesis of lipids. For the execution of these functions, membrane integrity is very important, and its disruption can directly or indirectly cause metabolic dysfunction eventually leads to bacterial death. Catechins (34) attract lipid bilayers of the membrane which involves the following mechanisms [137]. Catechins form hydrogen bonds, which attract polar head groups of lipids at the membrane edge. Epicatechin (35) and epigallocatechin gallate (26) alter phospholipids, which can alter structural changes in the cell membrane. Moreover, these catechins promote the inactivation or inhibition of intracellular and extracellular enzyme synthesis [137]. Generally, the inhibition of enzymes in fatty acid biosynthesis is an excellent target for antimicrobial agents for blocking bacterial growth, especially the key enzyme fatty acid synthase II (FAS-II) inhibitor is significant as an antimicrobial drug. Quercetin (1), apigenin (8), and sakuranetin (36) have been demonstrated to inhibit 3-hydroxyacyl-ACP dehydrase from *Helicobacter pylori* [138] and eriodictyol (37). Further, naringenin (3) and taxifolin (38) (Figure 2) inhibit 3-ketoacyl- ACP synthase from *E. faecalis* [139]. Flavonoids such as Epigallocatechin gallate (EGCG) (20), 5, 6, 7, 40, 50- pentahydroxyflavone (39), and 5-hydroxy-40, 7-dimethoxyflavone (40) inhibit the malonyl CoA-acyl carrier protein transacylase that regulates bacterial FAS-II [140,141]. EGCG (20) inhibits 3-ketoacyl-ACP reductase and enoyl-ACP reductase and prevents fatty acid biosynthesis [142]. Quercetin (1), kaempferol (23), 4, 20, 40-trihydroxychalcone (41), fisetin (42), morin (9), myricetin (43), baicalein (44), luteolin (45), EGCG (20), butein (46), and isoliquirtigenin (47) (Figure 2) inhibit various enzymes involved in fatty acid synthesis, including, FAS-II, enoyl-ACP-reductase, β-ketoacyl-ACP reductase, and β-hydroxy acyl-ACP dehydratases in *Mycobacterium sp*. [143]. Baicalein (44), EGCG (20), galangin (18), kaempferide (48), DL-cycloserine (49), quercetin (1), apigenin (8), and kaempferide-3-O-glucoside (50) (Figure 2) inhibit the synthesis of peptidoglycan, which is an essential component of the bacterial cell wall, resulting in cell wall damage [144,145,146]. 

### 4.4. Inhibition of Prokaryotic DNA Replication

Alkaloids are nitrogenous compounds characterized by their alkaline nature, which aids the inhibition of cell respiration, intercalates with DNA, and inhibits various enzymes involved in replication, transcription, and translation [147]. Plant-based bioactive compounds such as quercetin (1), nobiletin (51), myricetin (43), tangeritin (52,) genistein (31), apigenin (8), chrysin (24), kaempferol (23), and 3, 6, 7, 30, 40-pentahydroxyflavone (39) have been recognized as noteworthy DNA gyrase inhibitors, which are essential for DNA replication in prokaryotes including *V. harveyi, B. subtilis, M. smegmatis, M. tuberculosis*, and *E. coli* [146,148,149,150,151]. These bioactive compounds binding to the β subunit of gyrase and the corresponding blockage of the ATP binding pocket eventually contribute to the antimicrobial activity. Bioactive compounds have mediated the dysfunction of DNA gyrase functions in a dose-dependent manner that leads to the impairment of cell division and/or completion of chromosome replication, resulting in the inhibition of bacterial growth [149]. Luteolin (45), morin (9), and myricetin (43) have been demonstrated to inhibit the helicases of *E. coli* [152]. Helicases consititute another significant replicative enzyme responsible for separating and/or rearranging DNA double-strands [153]. Furthermore, myricetin (43) and baicalein (44) have been proposed as potent inhibitors of numerous DNA and RNA polymerases, as well as viral reverse transcriptase, resulting in the inhibition of bacterial growth [154]. EGCG (20), myricetin (43), and robinetin (53) have been demonstrated as inhibitors of dihydrofolate reductase in *Streptomonas maltophilia, P.vulgaris, S. aureus, M. tuberculosis*, and *E. coli* [43,155,156]. Dihydrofolate reductase is key enzyme for the synthesis of the purine and pyrimidine rings of nucleic acid, resulting in reduced DNA, RNA, and protein synthesis [156].

### 4.5. Inhibition of Energy Production

Energy production or ATP synthesis is the supreme vital requirement for the existence and development of bacteria as these chemicals are the main source of living systems. The treatment of flavonoids such as isobavachalcone (54) and 6-prenylapigenin (55) with *S. aureus* cause membrane depolarization, resulting in bacterial cell wall lysis [101]. Similarly, licochalcones (56) inhibited oxygen consumption in *M. luteus*, interruping the electron transport system eventually killing the bacteria [6]. It has been described that flavonoids such as baicalein (44), morin (9), silibinin (57), quercetin (1), isoquercetin (58), quercitrin (59), and silymarin (60) can constrain the F1FO ATPase system of *E. coli* and result in the obstruction of ATP synthesis [157,158,159]. Additionally, EGCG (20), 40, 50, 5-trihydroxy-6, 7-dimethoxy-flavone (61), and proanthocyanidins (27) have also inhibited *S. mutans, P. aeruginosa* and *S. aureus* through the enzymatic activity of F1FO ATPase respectively [100,104,141].

### 4.6. Inhibition of Bacterial Toxins

It is noteworthy that catechins and other flavonoids can cause bacterial cell wall destruction, resulting in an inability to discharge toxins [160,161]. Catechins (34), pinocembrin, kaempferol, EGCG (20), gallocatechin gallate (26), kaempferol-3-O-rutinoside (62), genistein (31), quercetin glycoside (63), and proanthocyanidins (27) (Figure 2) are suggested to neutralize bacterial toxic factors initiating from *V. cholerae, E. coli, S. aureus, V. vulnificus, B. anthracis, N. gonorrhoeae*, and *C. botulinum* [162,163,164,165]. Bacterial hyaluronidases are enzymes formed by both Gram-positive and Gram-negative bacteria and directly interact with host tissues, causing the permeability of connective tissues and reducing the viscosity of body fluids due to hyaluronidase-mediated degradation [166]. Flavonoids such as myricetin (43) and quercetin (1) have been identified as hyaluronic acid lyase inhibitors in *Streptococcus equisimilis* and *Streptococcus agalactiae* [167].

### 4.7. Mechanism of Resistance to Antibacterial Agents

Pathogenic bacteria generally receive the resistance to various antibiotics through diverse mechanisms. Such mechanisms include: (a) bacteria can share the resistance genes through transformation, transduction, and conjugation; (b) bacteria produce various enzymes to deactivate the antibiotics through the process of phosphorylation, adenylation, or acetylation; (c) damage or alteration of the drug compound; (c) prevent the interaction of the drug with the target; (d) efflux of the antibiotic from the cell [168,169,170]. Emodin (1, 2, 8-trihydroxy-6-methylanthraquinone) (64) is an anthraquinone derivative which prevents the transformation of resistance genes in *S. aureus* [171]. Baicalein is a potent inhibitor of the expression of the SOS genes, *RecA, LexA*, and *SACOL1400* that prevent rifampin-resistant mutation in *S. aureus* [172]. Phenolic compounds such as Carnosic (65) and rosmarinic acids (66) inactivate cmeB, cmeF, and cmeR genes in *Campylobacter jejuni* [173].

### 4.8. Antimicrobial Action with Generation of Reactive Oxygen Species

Reactive oxygen species (ROS) can be formed by the partial reduction of molecular oxygen that targets the exertion of antimicrobial activity, which aids host defense against various disease-causing pathogens. The suggested method of antimicrobial activity of catechins (34) involves augmentation of the production of oxidative stress (ROS and RNS), which can alter membrane permeability and cause as cell wall damage [174]. In addition, catechins damage liposomes as they contain a high amount of negatively charged lipids and are susceptible to damage [175]. An earlier study indicated that catechins support the leaking of potassium and disturbs the membrane transport system in a methicillin-resistant *S. aureus* strain [85]. This team has further demonstrated that acylated 3-O-octanoyl-epicatechin (21) is a lipophilic compound that produces more outcomes in antibacterial activity.

## 5. Conclusions

Since time immemorial, traditional medicinal plants have been cultivated by diverse populations to treat a great number of infectious diseases. Various investigations on the pharmacognostics and kinetics of medicinal plants have shown that crude extracts and plant-derived bioactive compounds may enhance the effects of traditional antimicrobials, which may be cost-effective, have fewer side effects, and improve the quality of treatment. Numerous studies have shown that the antimicrobial activity of plant extracts and their active compounds have the following potential: promote cell wall disruption and lysis, induce reactive oxygen species production, inhibit biofilm formation, inhibit cell wall construction, inhibit microbial DNA replication, inhibit energy synthesis, and inhibit bacterial toxins to the host. In addition, these compounds may prevent antibacterial resistance as well as synergetics to antibiotics, which can ultimately kill pathogenic organisms. Based on these comprehensive antimicrobial mechanisms, the cultivation of traditional plant extracts and bioactive compounds offers a promising treatment for disease-causing infectious microbial pathogens. Hence, this mechanism constitutes an encouraging ally in the development of pharmacological agents required to combat the growing number of microbial strains that have become resistant to extant antibiotics in clinical practice.

## Figures and Tables

**Table 1:** Antimicrobial screening performed on various medicinal plants.

Botanical Name	Family	Plant Used	Extracts	MIC *	Gram Positive	Gram Negative	Fungi	References
*Barleria prionitis* L.	Acanthaceae	Leaves	Pet. Ether	3.33–33.3 mg/mL	*B. subtilis, M. luteus, B. cereus, S. mutans, S. aureus, L. sporogenes*	*S. typhi, V. Cholera, M. luteus, Citrobacter*	-	[19]
			Chloroform	5–50 mg/mL	*B. subtilis, L. sporogenes*	*S. typhi, V. cholerae, Citrobacter, Providencia*	-	
			Methanol	10–100 mg/mL	*B. subtilis, L. sporogenes*	*V. cholerae, S. typhi,*	-	
			Ethanol	50–600 μg/mL	-	*S. typhi*	-	
		Bark	Acetone	25, 50, 100 mg/mL	*Bacillus spp., S. mutans, S. aureus,*	*Pseudomonas* spp.,	*S. cerevisiae, C. albicans*	
			Ethanol	25, 50, 100 mg/mL				
			Methanol	25, 50, 100 mg/mL				
*Adhatoda vasica* L.	Acanthaceae	Leaves	Aqueous	4% v/v	*M. tuberculosis,*	*E. coli, S. typhi*	-	[20]
			Methanol	625 µg/mL	*S. aureus*	*E. coli, S. typhi*	-	
*Pellaea calomelanos* L.	Adiantaceae	Leaves, Rhizomes	Aqueous,	250 μg/mL	*S. aureus, methicillin- resistant S. aureus, gentamycin– methicillin-resistant S. aureus, S. epidermidis, B. agri, P. acnes*	*P. aeruginosa*	*T. mentagrophytes, M. canis*	[21]
			Dichloromethane/Methanol	750–12,000 μg/mL				
*Sambucus australis* Cham. & Schltdl.	Adoxaceae	Leaves and Bark	Hexane	50 μg/mL	*S. aureus, S. agalactiae*	*E. coli*, *S. typhimurium* and *K. pneumoniae*	*C. albicans*	[22]
			Ethanol					
*Carpobrotus edulis* L N.E.Br.	Aizoaceae	Leaves	Aqueous	100 μg/mL	*S. mutans*, *S. sanguis*, *L. acidophilus L. casei*	*P. gingivalis F. nucleatum*	*C. albicans C. glabrataC. krusei*	[23]
			Dichloromethane/Methanol	750–12,000 μg/mL				
*Achyranthes aspera* L.	Amaranthaceae	Root, Leaves, Stem	Ethanol	1 mg/mL	*S. aureus, B. subtilis,*	*E. coli, P. vulgaris, K. pneumoniae*	-	[16]
*Alternanthera Sessile* L.	Amaranthaceae	Leaves	Ethanol	75 μg/mL	*S. pyogenes*	*S. typhi*	-	[24,25]
*Amaranthus caudatus* L.	Amaranthaceae	Leaves	Ethyl Acetate	162.2–665 mg/mL	*S. aureus, Bacillus spp.*	*E. coli, S. typhi, P. mirabilis*	-	[26]
			Chloroform	1.25 mg/mL				
			Methanol	3–5 mg/mL				
*Amaranthus hybridus* L.	Amaranthaceae	Leaves	Ethyl Acetate	200–755 mg/mL	-	*E. coli, S. typhi, k. pneumoniae, P. aeruginosa*	-	[26]
			Chloroform	1.25 mg/mL				
			Methanol	3–5 mg/mL				
*Amaranthus spinosus* L.	Amaranthaceae	Leaves	Ethyl Acetate	129 mg/mL	-	*S. typhi*	-	[26]
			Chloroform	1.25 mg/mL				
			Methanol	3–5 mg/mL				
*Boophane disticha* L.f.	Amaryllidaceae	Leaves	Aqueous,	20–100 mg/mL	*S. aureus, methicillin- resistant S. aureus, gentamycin– methicillin-resistant S. aureus, S. epidermidis, B. agri, P. acnes*	*P. aeruginosa*	*T. mentagrophytes, M. canis*	[21]
			Dichloromethane/Methanol	750–12,000 μg/mL				
*Scadoxus puniceus* (L.) Friis &Nordal.	Amaryllidaceae	Rhizomes, Roots	Aqueous	50 μg/mL	*S. aureus, methicillin- resistant S. aureus, gentamycin– methicillin-resistant S. aureus, S. epidermidis, B. agri, P. acnes*	*P. aeruginosa*	*T. mentagrophytes, M. canis*	[21]
			Dichloromethane/Methanol	750–12,000 μg/mL				
*Harpephyllum caffrum* Bernh. exKrauss	Anacardiaceae	Bark, Leaves	Aqueous	125–500 μg/mL	*S. aureus, methicillin- resistant S. aureus, gentamycin– methicillin-resistant S. aureus, S. epidermidis, B. agri, P. acnes*	*P. aeruginosa*	*T. mentagrophytes, M. canis*	[21]
			Dichloromethane/Methanol	750–12,000 μg/mL				
*Lannea discolor* Engl.	Anacardiaceae	Leaves	Aqueous	50–200 μg/mL	*S. aureus, methicillin- resistant S. aureus, gentamycin– methicillin-resistant S. aureus, S. epidermidis, B. agri, P. acnes*	*P. aeruginosa*	*T. mentagrophytes, M. canis*	[21]
			Dichloromethane/Methanol	750–12,000 μg/mL				
*Polyalthia cerascides* L.	Annonaceae	Stem Bark	Dichloromethane	100 μg/mL	*C. Dipthieriae*	-	-	[27]
*Berula erecta* Huds., Coville	Apiaceae	Rhizome, Leaves, Stem	Aqueous	2–16 μg/mL	*S. mutans*, *S. sanguis*, *L. acidophilus L. casei*	*P. gingivalis F. nucleatum*	*C. albicans C. glabrata C. krusei*	[23]
			Dichloromethane/Methanol	750–12,000 μg/mL				
*Acokanthera oppositifolia* L. Codd.	Apocynaceae	Leaves, Stem	Aqueous	25–200 μg/mL	*S. mutans*, *S. sanguis*, *L. acidophilus L. casei*	*P. gingivalis F. nucleatum*	*C. albicans C. glabrata C. krusei*	[23]
			Dichloromethane/Methanol	750–12,000 μg/mL				
*Plumeria ruba* L.	Apocynaceae	Leaves	Aqueous	50–200 μg/mL	*S. epidermidis*	*E. coli*	-	[16]
			Dichloromethane/Methanol	100 μg/mL				
*Acokanthera oppositifolia* (Laim.) Codd.,	Apocynaceae	Leaves	Aqueous	10–50 μg/mL	*S. aureus, methicillin- resistant S. aureus, gentamycin– methicillin-resistant S. aureus, S. epidermidis, B. agri, P. acnes*	*P. aeruginosa*	*T. mentagrophytes, M. canis*	[21]
			Dichloromethane/Methanol	750–12,000 μg/mL				
*Rauvolfia caffra* Sond.	Apocynaceae	Leaves	Aqueous	25, 50 μg/mL	*S. aureus, methicillin- resistant S. aureus, gentamycin– methicillin-resistant S. aureus, S. epidermidis, B. agri, P. acnes*	*P. aeruginosa*	*T. mentagrophytes, M. canis*	[21]
			Dichloromethane/Methanol	750–12,000 μg/mL				
*Calotropis gigantea* L.	Apocynaceae	Latex	Ethanol	1–8 mg/mL	-	-	*C. albicans, T. mentagrophytes, T. rubrum*	[16]
*Plumeria alba* L.	Apocynaceae	Root	Methanol	10–40 μg/mL	-	*E. coli*		[16]
*Ilex mitis* Radlk.	Aquifoliaceae	Bark, Leaves	Aqueous	1–8 mg/mL	*S. aureus, methicillin- resistant S. aureus, gentamycin– methicillin-resistant S. aureus, S. epidermidis, B. agri, P. acnes*	*P. aeruginosa*	*T. mentagrophytes, M. canis*	[21]
			Dichloromethane/Methanol	750–12,000 μg/mL				
*Anchomanes difformis* Engl.	Araceae	Roots	Methanol	20–100 mg/mL	methicillin-resistant *S. aureus*	-	-	[28]
*Zantedeschia aethiopica* Spreng	Araceae	Leaves	Aqueous	50 μg/mL	*S. aureus, methicillin- resistant S. aureus, gentamycin– methicillin-resistant S. aureus, S. epidermidis, B. agri, P. acnes*	*P. aeruginosa*	*T. mentagrophytes, M. canis*	[21]
			Dichloromethane/Methanol	15–150 μg/mL				
*Arum dioscoridis* L.	Araceae	Leaves	Aqueous	125–500 μg/mL	*S. aureus, S. pneumoniae*	*E. coli, S. typhi, P. aeruginosa*	-	[29]
*Aristolochia Indica* L.	Aristolochiaceae	Leaves	Ethanol	1–8 mg/mL	-	-	*A. niger A. flavus A. fumigatus*	[3,4,30,31]
*Vernonia blumeoides* Hook. f.	Asteraceae	Aerial Part	Ethanol	100 μg/mL	methicillin-resistant *S. aureus*	-	-	[28]
*Artemisia afra* Jacq. ex Willd.	Asteraceae	Leaves, Stem	Aqueous	2–16 μg/mL	*S. mutans*, *S. sanguis*, *L. acidophilus L. casei*	*P. gingivalis F. nucleatum*	*C. albicans C. glabrata C. krusei*	[23]
			Dichloromethane/Methanol	750–12,000 μg/mL				
*Tarchonanthus camphoratus* L.	Asteraceae	Leaves	Aqueous	25–200 μg/mL	*S. mutans*, *S. sanguis*, *L. acidophilus L. casei*	*P. gingivalis F. nucleatum*	*C. albicans C. glabrata C. krusei*	[23]
			Dichloromethane/Methanol	750–12,000 μg/mL				
*Helichrysum paronychioides* L.	Asteraceae	Whole Plant	Pet ether	50–200 μg/mL	*B. cereus*	*S. flexneri*	*C. glabrata*, *C. krusei*, *T. rubrum* and *T. tonsurans*	[2]
			Methanol	50–200 μg/mL				
*Senecio longiflorus* L.	Asteraceae	Stem and Leaves	Pet ether	125–625 μg/mL	*B. cereus*	*S. flexneri*	*C. glabrata*, *C. krusei*, *T. rubrum* and *T. tonsurans*	[2]
			Methanol	50–200 μg/mL				
*Dahlia pinnata* L.	Asteraceae	Leaves	Chloroform	2–16 μg/mL	*–*	*E. aerogenes, P. aeruginosa*	–	[16]
*Athrixia phylicoides* DC.	Asteraceae	Leaves	Aqueous	25–200 μg/mL	*S. aureus, methicillin- resistant S. aureus, gentamycin– methicillin-resistant S. aureus, S. epidermidis, B. agri, P. acnes*	*P. aeruginosa*	*T. mentagrophytes, M. canis*	[21]
			Dichloromethane/Methanol	750–12,000 μg/ml				
*Dicoma anomala* Sond.	Asteraceae	Tuber	Aqueous	50–200 μg/mL	*S. aureus, methicillin- resistant S. aureus, gentamycin– methicillin-resistant S. aureus, S. epidermidis, B. agri, P. acnes*	*P. aeruginosa*	*T. mentagrophytes, M. canis*	[21]
			Dichloromethane/Methanol	750–12,000 μg/mL				
*Vernonia natalensis* Sch. Bip. exWalp.	Asteraceae	Leaves, Roots	Aqueous	10–50 μg/mL	*S. aureus, methicillin- resistant S. aureus, gentamycin– methicillin-resistant S. aureus, S. epidermidis, B. agri, P. acnes*	*P. aeruginosa*	*T. mentagrophytes, M. canis*	[21]
			Dichloromethane/Methanol	750–12,000 μg/mL				
*Achillea millefolium* L.	Asteraceae	Leaves	Ethanol	100 μg/mL	*S. aureus*	*P. aeruginosa S. typhi, E. coli*	*C. albicans*	[29]
*Blumea balsamifer* (Linn.) D.C.	Asteraceae	Whole Plant	Ethanol	250 μg/mL	methicillin-resistant *S. aureus*	-	-	[32]
*Impatiens balsamina* L.	Balsaminaceae	Leaf	Ethanol	50–200 μg/ml	methicillin-resistant *S. aureus*	-	-	[28]
*Berberis chitria* L.	Berberidaceae	Roots	Ethanol,	5.5–6.5 mg/mL	*S. aureus*	*E. coli*	-	[33]
			Methanol	2.5–3.5 mg/mL				
*Alnus nepalensis* D. Don.	Betulaceae	TBL	Ethanol	50–200 μg/mL	Methicillin-resistant *S. aureus*	-	-	[32]
*Tecoma capensis* Lindl.	Bignoniaceae	Leaves, Stem	Aqueous,	10–50 μg/mL	*S. mutans*, *S. sanguis*, *L. acidophilus L. casei*	*P. gingivalis F. nucleatum*	*C. albicans C. glabrata C. krusei*	[23]
			Dichloromethane/Methanol	2.5 mg/mL				
*Spathodea campanulata* L.	Bignoniaceae	Leaves	Ethanol	221–254 μg/mL	*B. subtilis, S. aureus,*	*E. coli, K. pneumonia, P. vulgaris, S. typhi, Pseudomonas* spp., *V. cholerae*	-	[6,34,35]
		Flowers		156–173 μg/mL				
*Kigelia africana* (Lam.) Benth.	Bignoniaceae	Fruit	Aqueous	2–16 μg/mL	*S. aureus, methicillin- resistant S. aureus, gentamycin– methicillin-resistant S. aureus, S. epidermidis, B. agri, P. acnes*	*P. aeruginosa*	*T. mentagrophytes, M. canis*	[21]
			Dichloromethane/Methanol	750–12,000 μg/mL				
*Opuntia ficus-indica* Mill.	Cactaceae	Leaves	Aqueous	25–200 μg/mL	*S. aureus, methicillin- resistant S. aureus, gentamycin– methicillin-resistant S. aureus, S. epidermidis, B. agri, P. acnes*	*P. aeruginosa*	*T. mentagrophytes, M. canis*	[21]
			Dichloromethane	750–12,000 μg/mL				
			Methanol					
*Senna italic* L.	Caesalpiniaceae	Leaves	Acetone	2.5 mg/mL	*B. cereus, B. pumilus, B. subtilis, S. aureus, E. faecalis,*	-	-	[36]
*Cassia fistula* L.	Caesalpiniaceae	Seeds	Aqueous	780–6250 μg /mL	*S. aureus*	-	-	[6]
			Ethanol	2–16 μg/mL				
*Warburgia salutaris* (G. Bertol.) Chiov.	Canellaceae	Bark, Twigs	Aqueous	5.0–10 mg/mL	*S. mutans*, *S. sanguis*, *L. acidophilus L. casei*	*P. gingivalis F. nucleatum*	*C. albicans C. glabrata* *C. krusei*	[23]
				50–200 μg/mL	*S. aureus, methicillin- resistant S. aureus, gentamycin– methicillin-resistant S. aureus, S. epidermidis, B. agri, P. acnes*	*P. aeruginosa*	*T. mentagrophytes, M. canis*	[21]
			Dichloromethane, Methanol	750–12,000 μg/mL				
*Cadaba fruticosa* L.	Capparaceae	Leaves	Acetone	100–200 μg/mL	*S. pyogens, S. aureus, B. subtilis*	*S. typhi, P. vulgaris, K. pneumoniae, P. aeruginosa, E. coli*	-	[37]
			Aqueous	4–16 μg/mL				
			Benzene	4–16 μg/mL				
			Butanol	4–16 μg/mL				
			Chloroform	4–16 μg/mL				
			Ethanol	4–16 μg/mL				
*Boscia senegalensis* Del.	Capparidaceae	Roots	Methanol	10–20 μg/mL	methicillin-resistant *S. aureus*	-	-	[28]
*Celastrus orbiculatus* Thunb.	Celastraceae	Vane	Ethanol	1–2 mg/mL	methicillin-resistant *S. aureus*	-	-	[32]
*Euonymus fortunei* (Turcz.); Hand. Mazz.	Celastraceae	Leaves	Ethanol	10–40 μg/mL	methicillin-resistant *S. aureus*	-	-	[32]
*Chenopodium ambrosioides* Bert. ex Steud.	Chenopodiaceae	Leaves	Aqueous	2–16 μg/mL	*S. aureus, methicillin- resistant S. aureus, gentamycin– methicillin-resistant S. aureus, S. epidermidis, B. agri, P. acnes*	*P. aeruginosa*	*T. mentagrophytes, M. canis*	[21]
			Dichloromethane/Methanol	750–12,000 μg/mL				
*Garcinia mangostana L.*	Clusiaceae	Fruit Shell	Ethanol	25–200 μg/mL	methicillin-resistant *S. aureus*	-	-	[28]
*Garcinia morella* Desr.	Clusiaceae	Whole Plant	Ethanol	100–400 μg/mL	methicillin-resistant *S. aureus*	-	-	[32]
*Terminalia paniculata* L.	Combretaceae	Stem Bark	Ethyl Acetate	3.25, 3.5 mg/mL	*S. aureus, B. subtilis*	-	-	[38]
			Methanol	5–20 μg/mL				
*Terminalia sericea* Burch. ex DC.	Combretaceae	Roots	Aqueous	100–300 μg/mL	*S. aureus, methicillin- resistant S. aureus, gentamycin– methicillin-resistant S. aureus, S. epidermidis, B. agri, P. acnes*	*P. aeruginosa*	*T. mentagrophytes, M. canis*	[21]
			Dichloromethane/Methanol	750–12,000 μg/mL				
*Eupatorium odoratum* L.	Compositae	Leaves	Benzene	300–600 μg/mL	*B. cereus, S. aureus*	*E. coli, K. pneumoniae, V. cholerae*	*C. albicans*	[39]
			Aqueous	300–600 μg/mL				
			Acetone	300–600 μg/mL				
*Acmella paniculata* L.	Compositae	Whole Plant	Chloroform	15 μg/mL	-	*E. aerogenes*	-	[40]
			Pet. ether	5–15 μg/mL				
			Methanol	5–15 μg/mL				
*Cotyledon orbiculata* L.	Crassulaceae	Leaves	Aqueous	5–30 μg/mL	*S. mutans*, *S. sanguis*, *L. acidophilus L. casei*	*P. gingivalis F. nucleatum*	*C. albicans C. glabrata* *C. krusei*	[23]
			Dichloromethane	750–12,000 μg/mL				
			Methanol					
*Cotyledono rbiculata* Forssk.	Crassulaceae	Leaves	Aqueous	25–200 μg/mL	*S. aureus, methicillin- resistant S. aureus, gentamycin– methicillin-resistant S. aureus, S. epidermidis, B. agri, P. acnes*	*P. aeruginosa*	*T. mentagrophytes, M. canis*	[21]
			Dichloromethane/Methanol	750–12,000 μg/mL				
*Mormodica basalmina* L.	Cucurbitaceae	Whole Plant	Methanol	500 μg/mL	methicillin-resistant *S. aureus*	-	-	[28]
*Coccinia grandis* L.	Cucurbitaceae	Leaves	Aqueous	500 μg/mL	*B. cereus, S. aureus*	-	-	[41]
			Dichloromethane/Methanol	2 mg/mL				
*Luffa acyntangula* L.	Cucurbitaceae	Leaves	Aqueous	5 mg/mL	*B. cereus, S. aureus*	-	-	[41]
			Dichloromethane	2 mg/mL				
			Methanol					
*Mukia maderspatana* L.	Cucurbitaceae	Leaves	Aqueous	5 mg/mL	*B. cereus, S. aureus*	-	-	[41]
			Dichloromethane/Methanol	1 mg/mL				
*Trichosanthes cucumerina* L.	Cucurbitaceae	Leaves	Aqueous	5 mg/mL	*B. cereus, S. aureus*	-	-	[41]
			Dichloromethane/Methanol	1 mg/mL				
*Momordica balsami- na* L.	Cucurbitaceae	Leaves, Roots	Acetone	500 μg/mL	*B. cereus, B. pumilus, B. subtilis, S. aureus, E. faecalis*	*E. coli, E. cloaceae, K. pneumoniae, P. aeruginosa, S. marcescens*	-	[42]
*Carex prainii* C.B. Clarke	Cyperaceae	Whole Plant	Ethanol	15–45 μg/mL	methicillin-resistant *S. aureus*	-	-	[32]
*Dioscorea dregeana* T. Durand & Schinz.	Dioscoreaceae	Tuber	Aqueous	5–30 μg/mL	*S. aureus, methicillin- resistant S. aureus, gentamycin– methicillin-resistant S. aureus, S. epidermidis, B. agri, P. acnes*	*P. aeruginosa*	*T. mentagrophytes, M. canis*	[21]
			Dichloromethane/Methanol	750–12,000 μg/mL				
*Sansevieria hyacinthoides* L.	Dracaenaceae	Leaves, rhizome	Aqueous,	1–4 mg/mL	*S. mutans*, *S. sanguis*, *L. acidophilus L. casei*	*P. gingivalis F. nucleatum*	*C. albicans C. glabrata* *C. krusei*	[23]
			Dichloromethane/Methanol	750–12,000 μg/mL				
*Diospyros mespiliformis* Hochst. exA. DC.	Ebenaceae	Leaves	Aqueous	15–45 μg/mL	*S. aureus, methicillin- resistant S. aureus, gentamycin– methicillin-resistant S. aureus, S. epidermidis, B. agri, P. acnes*	*P. aeruginosa*	*T. mentagrophytes, M. canis*	[21]
			Dichloromethane/Methanol	750–12,000 μg/mL				
*Phyllanthus amarus* Schum. Thonn.	Euphorbiaceae	Whole Plant	Methanol	650–600 μg/mL	methicillin-resistant *S. aureus*	-	-	[28]
*Croton gratissimus* Burch.	Euphorbiaceae	Leaves, Stem,	Aqueous	5 mg/mL	*S. mutans*, *S. sanguis*, *L. acidophilus L. casei*	*P. gingivalis F. nucleatum*	*C. albicans C. glabrataC. krusei*	[23]
			Dichloromethane/Methanol	750–12,000 μg/mL				
*Spirostachys africana* Sond.	Euphorbiaceae	Leaves, Bark	Aqueous	490 μg/mL	*S. mutans*, *S. sanguis*, *L. acidophilus L. casei*	*P. gingivalis F. nucleatum*	*C. albicans C. glabrataC. krusei*	[23]
			Dichloromethane/Methanol	750–12,000 μg/mL				
*Acalypha indica* L.	Euphorbiaceae	Leaves	Aqueous	4% v/v	*M. tuberculosis*	-	-	[43]
*Bridelia micrantha* Baill.	Euphorbiaceae	Bark, Leaves	Aqueous	5 mg/mL	*S. aureus, methicillin- resistant S. aureus, gentamycin– methicillin-resistant S. aureus, S. epidermidis, B. agri, P. acnes*	*P. aeruginosa*	*T. mentagrophytes, M. canis*	[21]
			Dichloromethane/Methanol	750–12,000 μg/mL				
*Emblica officinalis* L.	Euphorbiaceae	Leaves	Benzene	350–600 μg/mL	*B. cereus, S. aureus*	*E. coli, K. pneumoniae, V. cholerae*	*C. albicans*	[39]
			Aqueous	300–600 μg/mL				
			Acetone	300–600 μg/mL				
*Hevea brasiliensis* L.	Euphorbiaceae	Leaves	Benzene	350–600 μg/mL	*B. cereus, S. aureus*	*E. coli, K. pneumoniae, V. cholerae*	*C. albicans*	[39]
			Aqueous	300–600 μg/mL				
			Acetone	300–600 μg/mL				
*Mallotus yunnanensis* Pax et. Hoffm.	Euphorbiaceae	Tender Branches & Leaves	Ethanol	8–256 μg/mL	methicillin-resistant *S. aureus*	-	-	[32]
*Acacia albida* Del.	Fabaceae	Stem Bark	Methanol	50 μg/mL	methicillin-resistant *S. aureus*	-	-	[28]
*Acacia catechu* (L. f.) Willd	Fabaceae	Wood	Ethanol	100 μg/mL	methicillin-resistant *S. aureus*	-	-	[28]
*Peltophorum ptercarpum* (DC.)	Fabaceae	Bark	Ethanol	4% v/v	methicillin-resistant *S. aureus*	-	-	[28]
*Acacia erioloba* Edgew.	Fabaceae	Bark and Leaves	Aqueous	1.56–3.12 mg/mL	*S. aureus, methicillin– resistant S. aureus, gentamycin– methicillin-resistant S. aureus, S. epidermidis, B. agri, P. acnes*	*P. aeruginosa*	*T. mentagrophytes, M. canis*	[21]
			Dichloromethane/Methanol	750–12,000 μg/mL				
*Dichrostachys cinerea* L.	Fabaceae	Stem	Aqueous	129 mg/mL	*S. mutans*, *S. sanguis*, *L. acidophilus L. casei*	*P. gingivalis F. nucleatum*	*C. albicans C. glabrata* *C. krusei*	[23]
			Dichloromethane/Methanol	750–12,000 μg/mL				
*Albizia odoratissima* (L.f.) Benth	Fabaceae	Leaves	Hexane	7.5–15 mg/mL	*S. aureus*	*K. pneumoniae*, *E. coli*, *P. aeruginosa*, *P. vulgaris*	-	[44]
			Chloroform	859–6875 μg/mL				
			Ethyl Acetate	136–546 μg/mL				
			Methanol	136–546 μg/mL				
*Prosopis juliflora* L.	Fabaceae	Pod	Chloroform	250 μg/mL	*M. luteus, S. aureus, S. mutans*	-	-	[36]
*Bauhinia macranthera* Benth. Ex Hemsl.	Fabaceae	Leaves	Aqueous,	1.56–3.12 mg/mL	*S. aureus, methicillin- resistant S. aureus, gentamycin– methicillin-resistant S. aureus, S. epidermidis, B. agri, P. acnes*	*P. aeruginosa*	*T. mentagrophytes, M. canis*	[21]
			Dichloromethane/Methanol	750–12,000 μg/mL				
*Erythrina lysistemon* Hutch.	Fabaceae	Leaves	Aqueous	4 mg/mL	*S. aureus, methicillin- resistant S. aureus, gentamycin– methicillin-resistant S. aureus, S. epidermidis, B. agri, P. acnes, S. mutans*, *S. sanguis*, *L. acidophilus L. casei*	*P. aeruginosa, P. gingivalis F. nucleatum*	*T. mentagrophytes, M. canis, C. albicans C. glabrata* *C. krusei*	[21]
			Dichloromethane/Methanol	750–12,000 μg/mL				
*Elephantorrhiza elephantina* (Burch.) Skeels	Fabaceae	Leaves, roots and rhizomes	Aqueous	1–4 mg/mL	*S. aureus, methicillin- resistant S. aureus, gentamycin– methicillin-resistant S. aureus, S. epidermidis, B. agri, P. acnes, B. cereus*	*P. aeruginosa, S. flexneri*	*T. mentagrophytes, M. canis, C. glabrata*, *C. krusei*, *T. rubrum* and *T. tonsurans*	[21]
			Dichloromethane/Methanol	750–12,000 μg/mL				
*Albizia lebbeck* L.	Fabaceae	Leaves	Benzene, Aqueous and Acetone	350–600 μg/mL	*B. cereus, S. aureus*	*E. coli, K. pneumoniae, V. cholera*	*C. albicans*	[39]
*Adenanthera pavonina* L.	Fabaceae	Leaves	Aqueous	5 mg/mL	*B. cereus, S. aureus*	-	-	[41]
			Dichloromethane/Methanol	60 μg mg/mL				
*Alysicarpus vaginalis* L.	Fabaceae	Leaves	Aqueous	5 mg/mL	*B. cereus, S. aureus*	-	-	[41]
			Dichloromethane/Methanol	2 mg/mL				
*Bauhinia acuminate* L.	Fabaceae	Leaves	Aqueous	5 mg/mL	*B. cereus, S. aureus*	-	-	[41]
			Dichloromethane/Methanol	50 μg mg/mL				
*Bauhinia purpurea* L.	Fabaceae	Leaves	Aqueous	5 mg/mL	*B. cereus, S. aureus*	-	-	[41]
			Dichloromethane/Methanol	1 mg/mL				
*Bauhinia racemose* L.	Fabaceae	Leaves, Stem Bark	Aqueous	500 μg/mL	*B. cereus, S. aureus*	-	-	[41]
			Dichloromethane/Methanol	500 μg/mL				
*Cassia alata* L.	Fabaceae	Leaves	Aqueous	250 μg/mL	*B. cereus, S. aureus*	-	-	[41]
			Dichloromethane/Methanol	500 μg/mL				
*Cassia auriculata* L.	Fabaceae	Leaves	Aqueous	1 mg/mL	*B. cereus, S. aureus*	-	-	[41]
			Dichloromethane/Methanol	4 mg/mL				
*Cassia fistula* L.	Fabaceae	Root Bark, Stem Bark	Aqueous	1–5 mg/mL	*B. cereus, S. aureus*	-	-	[41]
			Dichloromethane/Methanol	500–1000 μg/mL				
*Cassia tora* L.	Fabaceae	Leaves, Root Bark, Stem Bark	Aqueous	250–4000 μg/mL	*B. cereus, S. aureus*	-	-	[41]
			Dichloromethane/Methanol	1000–4000 μg/mL				
*Crotalaria retusa* L.	Fabaceae	Leaves	Aqueous	4 mg/mL	*B. cereus, S. aureus*	-	-	[41]
			Dichloromethane/Methanol	60 μg/mL				
*Crotalaria verrucosa* L.	Fabaceae	Leaves	Aqueous	1 mg/mL	*B. cereus, S. aureus*	-	-	[41]
			Dichloromethane/Methanol	1 mg/mL				
*Derris Scandens* L.	Fabaceae	Leaves	Aqueous	100 μg/mL	*B. cereus, S. aureus*	-	-	[41]
			Dichloromethane/Methanol	4 mg/mL				
*Desmodiumtriflorum* (L.) DC. var. majus Wight & Arn.	Fabaceae	Stem Bark	Aqueous	1 mg/mL	*B. cereus, S. aureus*	-	-	[41]
			Dichloromethane/Methanol	25 μg/mL				
*Erythuria variegate* L.	Fabaceae	Leaves, Stem Bark	Aqueous	1–5 mg/mL	*B. cereus, S. aureus*	-	-	[41]
			Dichloromethane/Methanol	250–1000 μg/mL				
*Indigofera tinctoria* L.	Fabaceae	Leaves	Aqueous	500 μg/mL	*B. cereus, S. aureus*	-	-	[41]
			Dichloromethane/Methanol	4 mg/mL				
*Mimosa pudica* L.	Fabaceae	Stem Bark	Aqueous	1–2 mg/mL	*B. cereus, S. aureus*	-	-	[41]
			Dichloromethane/Methanol	250–5000 μg/mL				
*Myroxylon balsamum* L.	Fabaceae	Leaves	Aqueous	1 mg/mL	*B. cereus, S. aureus*	-	-	[41]
			Dichloromethane/Methanol	500 μg/mL				
*Pterocarpus marsupium* Roxb.	Fabaceae	Leaves	Aqueous	4 mg/mL	*B. cereus, S. aureus*	-	-	[41]
			Dichloromethane/Methanol	250 μg/mL				
*Pterocarpus santalinus* L.	Fabaceae	Leaves	Aqueous	2 mg/mL	*B. cereus, S. aureus*	-	-	[41]
			Dichloromethane/Methanol	4 mg/mL				
*Saraca asoca* (Roxb.) Willd	Fabaceae	Leaves	Aqueous	120 μg/mL	*B. cereus, S. aureus*	-	-	[41]
			Dichloromethane/Methanol	5 mg/mL				
*Sesbania grandiflora* (L.) Poiret	Fabaceae	Stem Bark, Root Bark, Leaves	Aqueous,	2 mg/mL	*B. cereus, S. aureus*	-	-	[41]
			Dichloromethane/Methanol	100 μg/mL				
*Tamarindus indica* L.	Fabaceae	Leaves	Aqueous	250–500 μg/mL	*B. cereus, S. aureus*	-	-	[41]
			Dichloromethane/Methanol	60–100 μg/mL				
*Tephrosia purpurea* L. Pers.	Fabaceae	Leaves	Aqueous	5 mg/mL	*B. cereus, S. aureus*	-	-	[41]
			Dichloromethane/Methanol	5 mg/mL				
*Butea monosperma* L.	Fabaceae	Leaves	Aqueous	4 mg/mL	*B. cereus, S. aureus,* methicillin-resistant *S. aureus*	-	-	[41,45]
			Dichloromethane/Methanol	2 mg/mL				
			Ethanol	100–200 μg/mL				
*Senna alata*	Fabaceae	Leaf	Ethanol	100 μg/mL	methicillin-resistant *S. aureus*	-	-	[46]
*Quercus infectoria* Olivier	Fagaceae	Nutgalls	Ethanol	100–200 μg/mL	methicillin-resistant *S. aureus*	-	-	[16]
*Cyclobalanopsis austroglauca* Y.T. Chang	Fagaceae	TBL	Ethanol	8–256 μg/mL	methicillin-resistant *S. aureus*	-	-	[32]
*Scaevola spinescens* L.	Goodeniaceae	Aerial parts	Ethyl Acetate, Methanol	500 μg/mL	*S. pyogenes, S. aureus*	-	-	[38]
*Gunnera perpensa* L.	Gunneraceae	Leaves, Rhizome	Aqueous,	4 mg/mL	*S. aureus, methicillin- resistant S. aureus, gentamycin– methicillin-resistant S. aureus, S. epidermidis, B. agri, P. acnes*	*P. aeruginosa*	*T. mentagrophytes, M. canis*	[21]
			Dichloromethane/Methanol	750–12,000 μg/mL				
*Eucomis punctate* L’Her.	Hyacinthaceae	Leaves	Aqueous,	500 μg/mL	*S. mutans*, *S. sanguis*, *L. acidophilus L. casei*	*P. gingivalis F. nucleatum*	*C. albicans C. glabrata* *C. krusei*	[23]
			Dichloromethane/Methanol	750–12,000 μg/mL				
*Drimia sanguinea* L.	Hyacinthaceae	Bulb	Pet ether	18.75, 37.5, 300, 600, 1200 μg/mL	*B. cereus*	*S. flexneri*	*C. glabrata*, *C. krusei*, *T. rubrum* and *T. tonsurans*	[2]
*Hypoxis hemerocallidea* L.	Hypoxidaceae	Leaves	Pet ether	195–12,500 μg/mL	*B. cereus*	*S. flexneri*	*T. rubrum, T.urans, C. glabrata C. krusei*	[47]
			Methanol	390–3125 μg/mL				
*Curculigo orchioides* Gaertn.	Hypoxidaceae	Whole Plant	Ethanol	8–256 μg/mL	methicillin-resistant *S. aureus*	-	-	[32]
*Illicium simonsii* Maxim.	Illiciaceae	TBL	Ethanol	8–256 μg/mL	methicillin-resistant *S. aureus*	-	-	[32]
*Aristea ecklonii* Baker.	Iridaceae	Leaves and Roots	Aqueous	129 mg/mL	*S. aureus, methicillin- resistant S. aureus, gentamycin– methicillin-resistant S. aureus, S. epidermidis, B. agri, P. acnes*	*P. aeruginosa*	*T. mentagrophytes, M. canis*	[21]
			Dichloromethane/Methanol	750–12,000 μg/mL				
*Tetradenia riparia* Hochst.	Lamiaceae	Leaves, Stem	Aqueous	200–755 mg/mL	*S. mutans*, *S. sanguis*, *L. acidophilus L. casei*	*P. gingivalis F. nucleatum*	*C. albicans C. glabrata* *C. krusei*	[23]
			Dichloromethane/Methanol	750–12,000 μg/mL				
*Thymus vulgaris* L.	Lamiaceae	Leaves	Essential Oil	50 μg/mL	methicillin-resistant *S. aureus*	-	-	[48]
*Mentha aquatica* L.	Lamiaceae	Aerial Parts	Methanol	1.56–3.12 mg/mL	*S. aureus*	*E. coli, P. aeruginosa*, *S. heidelberg*, *K. pneumoniae, E. aerogenes, M. morganii*	-	[49]
			Chloroform	128 μg/mL				
			Acetone	32–128 μg/mL				
*Stachys guyoniana* Noë ex. Batt.	Lamiaceae	Leaves	*n*-Butanol	4 mg/mL	*S. aureus*	*E. coli, P. aeruginosa*, *S. heidelberg*, *K. pneumoniae, E. aerogenes, M. morganii*	-	[49]
			Ethyl Acetate	128 μg/mL				
			Chloroform	32–128 μg/mL				
*Ocimum basilicum* L.	Lamiaceae	Stem, leaves	Ethanol	1–4 mg/mL	*S. aureus*	-	-	[38]
*Ocimum gratissimum* L.	Lamiaceae	Leaves	Methanol	780–6250 μg/mL	*S. aureus*	*S. typhi, E. coli, S. paratyphi*	-	[38]
*Ocimum sanctum* L.	Lamiaceae	Whole Plant	Methanol	360 μg/mL	*S. aureus, S. saprophyticus*	*S. typhi, E. coli, S. paratyphi*	-	[6]
*Mentha longifolia* Huds.	Lamiaceae	Leaves	Aqueous	150, 300, 600 μg/mL	*S. aureus, methicillin- resistant S. aureus, gentamycin– methicillin-resistant S. aureus, S. epidermidis, B. agri, P. acnes*	*P. aeruginosa*	*T. mentagrophytes, M. canis*	[21]
			Dichloromethane/Methanol	750–12,000 μg/mL				
*Melissa officinalis* L.	Lamiaceae	Leaves	Ethanol	49 μg/mL	-	*K. pneumoniae*	-	[42]
*Ocimum americanum* L.	Lamiaceae	Leaves	Acetone	2.5 mg/mL	*B. cereus, B. pumilus, B. subtilis, S. aureus, E. faecalis*	-	-	[16]
*Machilus salicina* Hance.	Lauraceae	Tender Branches & Leaves	Ethanol	500 μg/mL	methicillin-resistant *S. aureus*	-	-	[32]
*Meliosma squamulata* Hance.	Lauraceae	TBL	Ethanol	1–4 mg/mL	methicillin-resistant *S. aureus*	-	-	[32]
*Sophora alopecuroides*	Leguminosae	Aerial Parts, Seeds	Ethanol	129 mg/mL	*B. subtilis, S. aureus, B. subtilis*	*P. aeruginosa*	-	[50]
*Acacia karroo* Hayne.	Leguminosae	Leaves, Stem	Aqueous,	200–755 mg/mL	*S. mutans*, *S. sanguis*, *L. acidophilus L. casei*	*P. gingivalis F. nucleatum*	*C. albicans C. glabrata* *C. krusei*	[23]
			Dichloromethane/Methanol	750–12,000 μg/mL				
*Acacia polyacantha* Willd.	Leguminosae	Leaves, Stem	Aqueous	50 μg/mL	*S. mutans*, *S. sanguis*, *L. acidophilus L. casei*	*P. gingivalis F. nucleatum*	*C. albicans C. glabrata* *C. krusei*	[23]
			Dichloromethane/Methanol	750–12,000 μg/mL				
*Dalbergia obovate* E. Mey.	Leguminosae	Leaves, stem	Aqueous	1.56–3.12 mg/mL	*S. mutans*, *S. sanguis*, *L. acidophilus L. casei*	*P. gingivalis F. nucleatum*	*C. albicans C. glabrata* *C. krusei*	[23]
			Dichloromethane/Methanol	750–12,000 μg/mL				
*Sophora jaubertii*	Leguminosae	Aerial Parts, Seeds	Ethanol	4 mg/mL	*B. subtilis, P. aeruginosa, S. aureus*	-	-	[38]
*Glycyrrhiza glabra* L.	Leguminosae	Leaves	Methanol	1–4 mg/mL	*K. kristinae*, *M. luteus*, *S. auricularis*, *B. megaterium*	*A. bohemicus*, *E. coli*	-	[51]
*Allium cepa* L.	Liliaceae	Bulb	Aqueous	780–6250 μg/mL	*M. tuberculosis*	-	-	[43]
*Allium sativum* L.	Liliaceae	Bulb	Aqueous	4% v/v	*M. tuberculosis*	-	-	[43]
*Allium vera* L.	Liliaceae	Gel	Aqueous	4% v/v	*M. tuberculosis*	-	-	[43]
*Lobelia nicotianaefolia* L.	Lobeliaceae	Root	Chloroform	129 mg/mL	*S. aureus*	*P. aeruginosa*	-	[39]
			Acetone	6 mg/mL				
			Ethanol	6 mg/mL				
*Woodfordia fruticose* L.	Lythraceae	Flower	Aqueous	200–755 mg/mL	*S. aureus, B. cereus*	*S. typhi, E. coli, S. dysenteriae. V. cholerae*	-	[37]
			Dichloromethane/Methanol	100 mg/mL				
*Manglietia hongheensis* Y.m Shui et. W.H. Chen.	Magnoliaceae	TBL	Ethanol	50 μg/mL	methicillin-resistant *S. aureus*	-	-	[32]
*Malva parviflora* L.	Malvaceae	Leaves	Aqueous	500 μg/mL	*S. aureus, methicillin- resistant S. aureus, gentamycin– methicillin-resistant S. aureus, S. epidermidis, B. agri, P. acnes*	*P. aeruginosa*	*T. mentagrophytes, M. canis*	[21]
			Dichloromethane/Methanol	750–12,000 μg/mL				
*Sida rhombifolia* L.	Malvaceae	Stem	Chloroform	162.2–665 mg/mL	*S. lutea, B. subtilis,*	*E. coli, Shigella shiga*	-	[38]
*Walsura robusta* L.	Meliaceae	Wood	Ethanol	250 μg/mL	methicillin-resistant *S. aureus*	-	-	[28]
*Swietenia mahagoni*	Meliaceae	Seed	Ethanol	500 μg/mL	methicillin-resistant *S. aureus*	-	-	[52]
*Azadirachta indica*	Meliaceae	LeavesStem	MethanolAqueous	1.56–3.12 mg/mL	*M. luteus* *S. aureus, S. pyogenes*	*P. vulgaris* *E. coli, P. aeruginosa*	--	[53]
*Ekebergia capensis* Sparrm.	Meliaceae	Bark, Leaves	Aqueous	1.59–25 mg/mL	*S. aureus, methicillin- resistant S. aureus, gentamycin– methicillin-resistant S. aureus, S. epidermidis, B. agri, P. acnes*	*P. aeruginosa*	*T. mentagrophytes, M. canis*	[21]
			Dichloromethane/Methanol	750–12,000 μg/mL				
*Trichilia emetica* Vahl	Meliaceae	Leaves	Aqueous	50–600 μg/mL	*S. aureus, methicillin- resistant S. aureus, gentamycin– methicillin-resistant S. aureus, S. epidermidis, B. agri, P. acnes*	*P. aeruginosa*	*T. mentagrophytes, M. canis*	[21]
			Dichloromethane/Methanol	750–12,000 μg/mL				
*Melia azedarach* L.	Meliaceae	Leaves	Methanol	3.33–33.3 mg/mL	*B. cereus, S. aureus*	*E. coli, P. aeruginosa*	*A. niger, A. flavus*, *F. oxysporum*, *R. stolonifer*	[16]
			Ethanol	500 μg/mL				
			Pet.ether	1.56–3.12 mg/mL				
			Aqueous	10–30 mg/mL				
*Melianthus comosus* Vahl.	Melianthaceae	Leaves	Aqueous	50 mg/mL	*S. aureus, methicillin- resistant S. aureus, gentamycin– methicillin-resistant S. aureus, S. epidermidis, B. agri, P. acnes,* methicillin-resistant *S. aureus*	*P. aeruginosa*	*T. mentagrophytes, M. canis*	[28]
			Dichloromethane/Methanol	4–64 mg/mL				
*Melianthus major* L.	Melianthaceae	Leaves	Ethanol	10–100 mg/mL	methicillin-resistant *S. aureus*	-	-	[28]
*Melianthus major* L.	Melianthaceae	Leaves	Aqueous	5–50 mg/mL	*S. aureus, methicillin- resistant S. aureus, gentamycin– methicillin-resistant S. aureus, S. epidermidis, B. agri, P. acnes*	*P. aeruginosa*	*T. mentagrophytes, M. canis*	[21]
			Dichloromethane/Methanol	750–12,000 μg/mL				
*Cissampelos torulosa* E. Mey. Ex Harv.	Menispermaceae	Leaves, Stem	Aqueous	25, 50, 100 mg/mL	*S. mutans*, *S. sanguis*, *L. acidophilus L. casei*	*P. gingivalis F. nucleatum*	*C. albicans C. glabrata* *C. krusei*	[23]
			Dichloromethane/Methanol	750–12,000 μg/mL				
*Tinospora crispa* L.	Menispermaceae	Stem	Ethanol	10 mg/mL	methicillin-resistant *S. aureus*	-	-	[21]
*Cissampelos capensis* Thunb.	Menispermaceae	Leaves	Aqueous	3.33–33.3 mg/mL	*S. aureus, methicillin- resistant S. aureus, gentamycin– methicillin-resistant S. aureus, S. epidermidis, B. agri, P. acnes*	*P. aeruginosa*	*T. mentagrophytes, M. canis*	[21]
			Dichloromethane/Methanol	750–12,000 μg/mL				
*Ficus natalensis* Hochst.	Moraceae	Leaves	Aqueous	250 mg/mL	*S. aureus, methicillin- resistant S. aureus, gentamycin– methicillin-resistant S. aureus, S. epidermidis, B. agri, P. acnes*	*P. aeruginosa*	*T. mentagrophytes, M. canis*	[21]
			Dichloromethane/Methanol	750–12,000 μg/mL				
*Ficus sur* Forssk.	Moraceae	Bark, Leaves	Aqueous,	10–100 mg/mL	*S. aureus, methicillin- resistant S. aureus, gentamycin– methicillin-resistant S. aureus, S. epidermidis, B. agri, P. acnes*	*P. aeruginosa*	*T. mentagrophytes, M. canis*	[21]
			Dichloromethane/Methanol	750–12,000 μg/mL				
*Moringa oleifera* Lam.	Moringacceae	Leaf	Ethanol	5–50 mg/mL	methicillin-resistant *S. aureus*	-	-	[28]
*Myrothamnus flabellifolia* Welw.,	Myrothamnaceae	Leaves	Aqueous	156–625 μg/mL	*S. mutans*, *S. sanguis*, *L. acidophilus L. casei*	*P. gingivalis F. nucleatum*	*C. albicans C. glabrata* *C. krusei*	[23]
			Dichloromethane/Methanol	750–12,000 μg/mL				
*Embelia ruminate* (E. Mey.exA.Dc.) Mez	Myrsinaceae	leaves	Aqueous	350–600 μg/mL	*S. aureus, methicillin- resistant S. aureus, gentamycin– methicillin-resistant S. aureus, S. epidermidis, B. agri, P. acnes*	*P. aeruginosa*	*T. mentagrophytes, M. canis*	[21]
			Dichloromethane/Methanol	750–12,000 μg/mL				
*Embelia burm* f.	Myrsinaceae	Leaves	Ethanol	500 μg/mL	methicillin-resistant *S. aureus*	-	-	[32]
*Callistemon rigidus* R.Br.	Myrtaceae	Leaf	Methanol	800 mg/disc	methicillin-resistant *S. aureus*	-	-	[28]
*Psidium guajava* L.	Myrtaceae	Leaf	Ethanol	600, 1200 μg/mL	methicillin-resistant *S. aureus*	-	-	[28]
*Heteropyxis natalenesis* Harv.	Myrtaceae	Leaves, Stem	Aqueous,	5 mg/mL	*S. mutans*, *S. sanguis*, *L. acidophilus L. casei*	*P. gingivalis F. nucleatum*	*C. albicans C. glabrata* *C. krusei*	[23]
			Dichloromethane/Methanol	750–12,000 μg/mL				
*Eucalyptus camaldulensis* Dehnh.	Myrtaceae	Bark	Aqueous	9.375, 18.75, 37.5, 75, 150, 300, 600 μg/mL	*S. aureus, methicillin- resistant S. aureus, gentamycin– methicillin-resistant S. aureus, S. epidermidis, B. agri, P. acnes*	*P. aeruginosa*	*T. mentagrophytes, M. canis*	[21]
			Dichloromethane/Methanol	750–12,000 μg/mL				
*Eucalyptus deglupta*	Myrtaceae	Leaves	Benzene	37.5, 75, 150, 300, 600 μg/mL	*B. cereus, S. aureus*	*E. coli, K. pneumoniae, V. cholerae*	*C. albicans*	[39]
			Aqueous	4–8 mg/mL				
			Acetone	6 mg/mL				
*Myrtus communis* L.	Myrtaceae	Leaves	Ethanol	12.5–50 mg/mL	*B. cereus, L. monocytogenes*	*E. coli*	*C. albicans*	[42]
*Nelumbo nucifera* L.	Nelumbonaceae	Flower	Ethanol	8–32 mg/mL	*B. subtilis, S. aureus,*	*E. coli, K. pneumonia, P. aeruginosa*	-	[54]
*Nymphaea lotus* L.	Nymphaeaceae	Leaf	Ethanol	500 μg/mL	methicillin-resistant *S. aureus*	-	-	[21]
*Oxalis corniculata* L.	Oxalidaceae	Leaves	Aqueous	5 mg/mL	*B. cereus, S. aureus*	*E. coli, K. pneumoniae, V. cholera*	*C. albicans*	[39]
			Benzene	37.5, 75, 150, 300, 600 μg/mL				
			Acetone	6 mg/mL				
*Paeonia lactiflora* Pall.	Paeoniaceae	Leaves	Ethanol	22.4–52.3 μg/mL	*K. kristinae*, *M. luteus*, *S. auricularis*, *B. megaterium*	*A. bohemicus*, *E. coli*	-	[51]
*Argemone mexicana*	Papaveraceae	Stem	Chloroform	32.4–55.8 μg/mL	*S. aureus*	*E. coli, P. aeruginosa, k. pneumoniae*	-	[55]
*Passiflora Mexicana* L.	Passifloraceae	Aerial Parts	Ethanol	33.7–58.3 μg/mL	*S. aureus*	-	-	[21]
*Cleistanthus collinus*	Phyllanthaceae	Leaves	Benzene	100 mg/mL	*B. cereus, S. aureus*	*E. coli, K. pneumoniae, V. cholerae*	*C. albicans*	[39]
			Aqueous	4–8 mg/mL				
			Acetone	5 mg/mL				
*Piper nigrum* L.	Piperaceae	Bark, Seeds	Ethanol	500 μg/mL	*S. aureus, B. cereus, S. fecalis*	*P. aeruginosa, E. coli, S. typhi*	-	[38]
			Acetone	6 mg/mL				
			Dichloromethane/Methanol	12.5–50 μg/mL				
*Pittosporum viridiflorum* Sims.	Pittosporaceae	Leaves	Aqueous	600 μg/mL	*S. aureus, methicillin- resistant S. aureus, gentamycin– methicillin-resistant S. aureus, S. epidermidis, B. agri, P. acnes*	*P. aeruginosa*	*T. mentagrophytes, M. canis*	[21]
			Dichloromethane/Methanol	750–12,000 μg/mL				
*Spinifex littoreus*	Poaceae	Grass	Acetone	2.5 mg/mL	-	-	Dermatophytes	[27]
*Polygonum molle* D. Don.	Polygonaceae	Whole Plant	Ethanol	25–50 μg/mL	Methicillin-resistant *S. aureus*	-	-	[32]
*Eichhornia crassipes* L.	Pontederiaceae	Leaves, Shoot	Ethanol	500–4000 μg/mL	*M. luteus*	*R. rubrum*	*M. ruber, A. fumigates*	[56]
			Chloroform	32.4–55.8 μg/mL				
			Aqueous	2.5–15 μg/mL				
*Punica granatum* L.	Punicaceae	Fruit Shell	Ethanol	70 mg/mL	Methicillin-resistant *S. aureus*	-	-	[28]
*Clematis brachiate* Thunb.	Ranunculaceae	Flower, Leaves, Stem, Root	Aqueous,	1 mg/mL	*S. mutans*, *S. sanguis*, *L. acidophilus L. casei*	*P. gingivalis F. nucleatum*	*C. albicans C. glabrata* *C. krusei*	[23]
			Dichloromethane/Methanol	750–12,000 μg/mL				
*Ziziphus mucronata* Willd.	Rhamnaceae	Bark, Leabes	Aqueous	2.5 mg/mL	*S. aureus, methicillin- resistant S. aureus, gentamycin– methicillin-resistant S. aureus, S. epidermidis, B. agri, P. acnes, S. mutans*, *S. sanguis*, *L. acidophilus L. casei*	*P. aeruginosa, P. gingivalis F. nucleatum*	*T. mentagrophytes, M. canis, C. albicans C. glabrata C. krusei*	[21]
			Dichloromethane/Methanol	750–12,000 μg/mL				
*Eriobotrya japonica* (Thunb.) Lindl.	Rosaceae	Leaves	Ethanol	2–16 μg/mL	*K. kristinae*, *M. luteus*, *S. auricularis*, *B. megaterium*	*A. bohemicus*, *E. coli*	-	[51]
*Pavetta crassipes* K. Schum.	Rubiaceae	Leaf	Methanol	12.5–50 mg/mL	methicillin-resistant *S. aureus*	-	-	[28]
*Uncaria gambir* (Hunter) Roxb.	Rubiaceae	Leaf, Stem	Ethanol	8–32 mg/mL	methicillin-resistant *S. aureus*	-	-	[28]
*Vangueria spinose* L.	Rubiaceae	Leaves	Ethyl Acetate	500 μg/mL	*S. aureus*	*E. coli, K. pneumoniae, P. aeruginosa*	-	[57]
*Pentanisia prunelloides* Walp.	Rubiaceae	Root Bark	Aqueous	5 mg/mL	*S. aureus, methicillin- resistant S. aureus, gentamycin– methicillin-resistant S. aureus, S. epidermidis, B. agri, P. acnes*	*P. aeruginosa*	*T. mentagrophytes, M. canis*	[21]
			Dichloromethane/Methanol	750–12,000 μg/mL				
*Rothmannia capensis* Thunb.	Rubiaceae	Leaves	Aqueous	22.4–52.3 μg/mL	*S. aureus, methicillin- resistant S. aureus, gentamycin– methicillin-resistant S. aureus, S. epidermidis, B. agri, P. acnes*	*P. aeruginosa*	*T. mentagrophytes, M. canis*	[21]
			Dichloromethane/Methanol	750–12,000 μg/mL				
*Geophila repens* L.	Rubiaceae	Leaves, Stem Bark	Aqueous	1 mg/mL	*B. cereus, S. aureus*	-	-	[41]
			Dichloromethane/methanol	250 μg/mL				
*Guettarda speciose* L.	Rubiaceae	Leaves	Aqueous	2 mg/mL	*B. cereus, S. aureus*	-	-	[41]
			Dichloromethane/Methanol	2 mg/mL				
*Haldina cordifolia* L.	Rubiaceae	Leaves	Aqueous	1 mg/mL	*B. cereus, S. aureus*	-	-	[41]
			Dichloromethane/Methanol	500 μg/mL				
*Hedyotis auricularia* L.	Rubiaceae	Leaves	Aqueous	300 μg/mL	*B. cereus, S. aureus*	-	-	[41]
			Dichloromethane/Methanol	250 μg/mL				
*Knoxia zeylanica* L.	Rubiaceae	Leaves, Stem	Aqueous	250 μg/mL	*B. cereus, S. aureus*	-	-	[41]
			Dichloromethane/Methanol	1 mg/mL				
*Mitragyna parvifolia* L.	Rubiaceae	Leaves	Aqueous	300 μg/mL	*B. cereus, S. aureus*	-	-	[41]
			Dichloromethane/Methanol	1 mg/mL				
*Morinda umbellate* L.	Rubiaceae	Leaves, Stem Bark	Aqueous	100 μg/mL	*B. cereus, S. aureus*	-	-	[41]
			Dichloromethane/Methanol	250 μg/mL				
*Nauclea orientalis* L.	Rubiaceae	Leaves	Aqueous	500 μg/mL	*B. cereus, S. aureus*	-	-	[41]
			Dichloromethane/Methanol	500 μg/mL				
*Oldenlandia biflora* L.	Rubiaceae	Leaves	Aqueous	2 mg/mL	*B. cereus, S. aureus*	-	-	[41]
			Dichloromethane/Methanol	5 mg/mL				
*Oldenlandia herbacea* L.	Rubiaceae	Stem, Root	Aqueous	5mg/mL	*B. cereus, S. aureus*	-	-	[41]
			Dichloromethane/Methanol	60 μg/mL				
*Ophiorrhiza mungos* L.	Rubiaceae	Leaves	Aqueous	2 mg/mL	*B. cereus, S. aureus*	-	-	[41]
			Dichloromethane/Methanol	500 μg/mL				
*Paederia foetida* L.	Rubiaceae	Leaves, Stem	Aqueous	300 μg/mL	*B. cereus, S. aureus*	-	-	[41]
			Dichloromethane/Methanol	60 μg/mL				
*Pavetta lanceolate* Eckl.	Rubiaceae	Leaves	Aqueous	1 mg/mL	*B. cereus, S. aureus*	-	-	[41]
			Dichloromethane/Methanol	250 μg/mL				
*Spermacoce hispida* L.	Rubiaceae	Leaves, Stem	Aqueous	300 μg/mL	*B. cereus, S. aureus*	-	-	[41]
			Dichloromethane/Methanol	120 μg/mL				
*Wendlandia bicuspidate* Wight & Arn.	Rubiaceae	Leaves	Aqueous	60 μg/mL	*B. cereus, S. aureus*	-	-	[41]
			Dichloromethane/Methanol	5 mg/mL				
*Chassalia kolly*	Rubiaceae	Whole Plant	Mthanol	5 mg/mL	*S. aureus*	*E. coli, P. aeruginosa, S. typhi, P. aeruginosa*	-	[16]
*Randia dumetorum* L.	Rubiaceae	Fruits	Methanol	9.375, 18.75, 37.5, 75, 150, 300, 600 μg/mL	*S. aureus, S. epidermidis, B. subtilis*	*E. coli, S. typhi*	-	[23]
*Mitragyna speciosa* L.	Rubiaceae	Leaves	Methanol	37.5, 75, 150, 300, 600 μg/mL	*S. typhi*			[42]
*Clausena anisate* (Willd) Hook. f. ex.	Rutaceae	Leaves, Stem, Twigs	Aqueous	12.5–50 mg/mL	*S. mutans*, *S. sanguis*, *L. acidophilus L. casei*	*P. gingivalis F. nucleatum*	*C. albicans C. glabrata C. krusei*	[23]
			Dichloromethane/Methanol	750–12,000 μg/mL				
*Zanthoxylum capense* Harv.	Rutaceae	Stem	Aqueous	8–32 mg/mL	*S. mutans*, *S. sanguis*, *L. acidophilus L. casei*	*P. gingivalis F. nucleatum*	*C. albicans C. glabrata C. krusei*	[23]
			Dichloromethane/Methanol	750–12,000 μg/mL				
*Aegle marmelos* L.	Rutaceae	Leaves and Fruits	Methanol	500 μg/ml	*S.aureus, B. cereus*	*E. coli, S. typhi, P. aeruginosa, S. boydii, K. aerogenes, P.vulgaris,*		[20]
*Evodia daneillii* (Benn) Hemsl.	Rutaceae	Tender Branches & Leaves	Ethanol	3.33–33.3 mg/mL	Methicillin-resistant *S. aureus*	-	-	[32]
*Skimmia arborescens* Anders.	Rutaceae	TBL	Ethanol	250 mg/mL	Methicillin-resistant *S. aureus*	-	-	[32]
*Salvadora australis*	Salvadoraceae	Leaves	Acetone	10–100 mg/mL	*B. cereus, B. pumilus, B. subtilis, S. aureus, E. faecalis*	-	-	[18]
*Viscum capense* L.f.	Santalaceae	Leaves	Aqueous	5–50 mg/mL	*S. aureus, methicillin- resistant S. aureus, gentamycin– methicillin-resistant S. aureus, S. epidermidis, B. agri, P. acnes*	*P. aeruginosa*	*T. mentagrophytes, M. canis*	[21]
			Dichloromethane/Methanol	750–12,000 μg/mL				
*Dodonaea angustifolia* (L.f.) Benth	Sapindaceae	Leaves	Ethanol	156–625 μg/mL	methicillin-resistant *S. aureus*	-	-	[28]
*Dodonaea viscosa* Jacq.	Sapindaceae	Leaves, Stem	Aqueous	350–600 μg/mL	*S. mutans*, *S. sanguis*, *L. acidophilus L. casei*	*P. gingivalis F. nucleatum*	*C. albicans C. glabrata* *C. krusei*	[23]
			Dichloromethane/Methanol	750–12,000 μg/mL				
*Cardiospermum halicacabum* L.	Sapindaceae	Leaves	*n*-Butanol	500 μg/mL	*S. aureus, S. agalactiae*	*E. coli*, *S. typhimurium* and *K. pneumoniae*	*T. rubrum, C. albicans*	[58]
			Ethyl acetate	60 μg/mL				
			Chloroform	40 μg/mL				
*Dodonaea angustifolia* L. f.	Sapindaceae	Leaves	Aqueous	800 mg/disc	*S. aureus, methicillin- resistant S. aureus, gentamycin– methicillin-resistant S. aureus, S. epidermidis, B. agri, P. acnes*	*P. aeruginosa*	*T. mentagrophytes, M. canis*	[21]
			Dichloromethane/Methanol	750–12,000 μg/mL				
*Englerophytum magalismontanum* Sonder.	Sapotaceae	Leaves, Stem	Aqueous	600, 1200 μg/mL	*S. mutans*, *S. sanguis*, *L. acidophilus L. casei*	*P. gingivalis F. nucleatum*	*C. albicans C. glabrataC. krusei*	[23]
			Dichloromethane/Methanol	750–12,000 μg/mL				
*Schisandra viridis* A.c. Smith.	Schisandraceae	Vane	Ethanol	5 mg/mL	Methicillin-resistant *S. aureus*	-	-	[32]
*Halleria lucida* L.	Scrophulariaceae	Leaves Stem	Aqueous	1–8 mg/mL	*S. aureus, methicillin- resistant S. aureus, gentamycin– methicillin-resistant S. aureus, S. epidermidis, B. agri, P. acnes*	*P. aeruginosa*	*T. mentagrophytes, M. canis*	[21]
			Dichloromethane/Methanol	750–12,000 μg/mL				
Brandisia hancei Hook.f.	Scrophulariaceae	Whole Plant	Ethanol	3.33–33.3 mg/mL	Methicillin-resistant *S. aureus*	-	-	[32]
*Selaginella tamariscina* (Seauv.) Spring.	Selaginellaceae	Whole Plant	Ethanol	250 mg/mL	Methicillin-resistant *S. aureus*	-	-	[32]
*Datura stramonium* L.	Solanaceae	Leaves, Stem, Fruit	Aqueous	10–100 mg/mL	*S. mutans*, *S. sanguis*, *L. acidophilus L. casei*	*P. gingivalis F. nucleatum*	*C. albicans C. glabrata* *C. krusei*	[23]
			Dichloromethane/Methanol	750–12,000 μg/mL				
*Solanum incanum* L	Solanaceae	Leaves	Aqueous	5–50 mg/mL	*S. aureus, methicillin- resistant S. aureus, gentamycin– methicillin-resistant S. aureus, S. epidermidis, B. agri, P. acnes*	*P. aeruginosa*	*T. mentagrophytes, M. canis*	[21]
			Dichloromethane/Methanol	750–12,000 μg/mL				
*Solanum trilobatum* L.	Solanaceae	Leaves	Acetone	156–625 μg/mL	*S. pyogens, S. aureus, B. subtilis*	*S. typhi, P. vulgaris, K. pneumoniae, P. aeruginosa, E. coli*	-	[37]
			Aqueous	250 mg/mL				
			Benzene	10–100 mg/mL				
			Butanol	5–50 mg/mL				
			Chloroform	60 μg/mL				
			Ethanol	5 mg/mL				
*Datura metel* L.	Solanaceae	Leaves	Aqueous	350–600 μg/mL	*B. cereus, S. aureus*	-	-	[41]
			Dichloromethane/Methanol	1 mg/mL				
*Solanum macrocarpon* L.	Solanaceae	Leaves, Stem	Aqueous	500 μg/mL	*B. cereus, S. aureus*	-	-	[41]
			Dichloromethane/Methanol	60 μg/mL				
*Solanum melongena* L.	Solanaceae	Leaves, Root Stem	Aqueous	800 mg/disc	*B. cereus, S. aureus*	-	-	[41]
			Dichloromethane/Methanol	100 μg/mL				
*Solanum nigrum* L.	Solanaceae	Leaves, Stem	Aqueous	600, 1200 μg/mL	*B. cereus, S. aureus*	-	-	[41]
			Dichloromethane/Methanol	1 mg/mL				
*Solanum torvum* Sw.	Solanaceae	Leaves	Aqueous	3.33–33.3 mg/mL	*B. cereus, S. aureus*	-	-	[41]
			Dichloromethane/Methanol	60 μg/mL				
*Solanum virginianum* L.	Solanaceae	Leaves, Stem, Root	Aqueous	250 mg/mL	*B. cereus, S. aureus*	-	-	[41]
			Dichloromethane/Methanol	4 mg/mL				
*Withania somnifera* (L.) Dunal	Solanaceae	Roots & Leaves	Aqueous	10–100 mg/mL	*B. cereus, S. aureus,* methicillin-resistant *S. aureus*	-	-	[41,59]
			Dichloromethane/Methanol	1 mg/mL				
*Cola acuminate* L.	Sterculiaceae	Stem	Acetone	5–50 mg/mL	*S. aureus*	-	*C. albicans*	[16]
			Methanol	100 μg/mL				
*Schima sinensis* (Hemsl. et. Wils) Airy-shaw.	Theaceae	Tbl	Ethanol	156–625 μg/mL	methicillin-resistant *S. aureus*	-	-	[32]
*Coriandrum sativum*	Umbelliferae	Seeds	Aqueous	350–600 μg/mL	*S. aureus*	*K. pneumoniae, P. aeruginosa,*	*A. niger, P. lilacinum*	[27]
*Clerodendrum inerme L*	Verbenaceae	Leaves	Methanol	500 μg/mL	*S. aureus*	-	*A. niger*	[60]
*Lantana rugosa* Thunb.	Verbenaceae	Leaves	Aqueous	800 mg/disc	*S. aureus, methicillin- resistant S. aureus, gentamycin– methicillin-resistant S. aureus, S. epidermidis, B. agri, P. acnes*	*P. aeruginosa*	*T. mentagrophytes, M. canis*	[21]
			Dichloromethane/Methanol	750–12,000 μg/mL				
*Lantana camara* L.	Verbenaceae	Leaves, Flower	Chloroform	600, 1200 μg/mL	*S. aureus, B. cereus*	*E. coli, S. typhi, P. aeruginosa, K. aerogenes, P. vulgaris, S. Boydii, K. pneumoniae, V. cholerae*	*A. fumigatus, A. flavus, A. niger, C. albicans*	[39]
			Acetone	5 mg/mL				
			Methanol	1–8 mg/mL				
			Aqueous	1–2 mg/mL				
*Lantana indica* L.	Verbenaceae	Leaves	Methanol	3.33–33.3 mg/mL	*B. subtilis, S. aureus, S. pyogenes,*	*E. coli, P. vulgaris, K. pneumoniae*	*C.* *albicans,*	[61]
			Aqueous	4 mg/mL				
*Cyphostemma lanigerum* Harv.	Vitaceae	Leaves, Stem	Aqueous	250 mg/mL	*S. mutans*, *S. sanguis*, *L. acidophilus L. casei*	*P. gingivalis F. nucleatum*	*C. albicans C. glabrata* *C. krusei*	[23]
			Dichloromethane/Methanol	750–12,000 μg/mL				
*Cyphostemma setosum* Roxb.	Vitaceae	Leaves, Stem, Fruit	Aqueous	10–100 mg/mL	*S. mutans*, *S. sanguis*, *L. acidophilus L. casei*	*P. gingivalis F. nucleatum*	*C. albicans C. glabrata C. krusei*	[23]
			Dichloromethane/Methanol	750–12,000 μg/mL				
*Aloe arborescens* Mill.	Xanthorrhoeaceae	Leaves	Aqueous	5–50 mg/mL	*S. aureus, methicillin- resistant S. aureus, gentamycin– methicillin-resistant S. aureus, S. epidermidis, B. agri, P. acnes*	*P. aeruginosa*	*T. mentagrophytes, M. canis*	[21]
			Dichloromethane/Methanol	750–12,000 μg/mL				
*Siphonochilus aethiopicus* Schweinf.,	Zingiberaceae	Leaves, Stem, Root	Aqueous	156–625 μg/mL	*S. mutans*, *S. sanguis*, *L. acidophilus L. casei*	*P. gingivalis F. nucleatum*	*C. albicans C. glabrata C. krusei*	[23]
			Dichloromethane/Methanol	750–12,000 μg/mL				
*Curcuma xanthorrhiza*	Zingiberaceae	Rhizome	Ethanol	350–600 μg/mL	methicillin-resistant *S. aureus*	-	-	[46]
*Kaempferia pandurata* Roxb.	Zingiberaceae	Rhizome	Ethanol	500 μg/mL	methicillin-resistant *S. aureus*	-	-	[46]
*Peganum harmala* L.	Zygophyllaceae	Seeds	Ethanol	800 mg/disc	*S. aureus*	*E. coli*	-	[21]

* MIC (minimum inhibitory concentration) is the lowest drug concentration at which a given antimicrobial extract inhibits the visible growth of a tested organism. **MIC absolute value**: the given absolute value of drug concentration inhibits the growth of all tested organisms/ **MIC ranges**: the given range of drug concentrations (minimum to maximum) inhibit the growth of the individual to all tested organisms.

**Table 2:** Antimicrobial activities of bioactive compounds.

Botanical Name	Family	Extracts	Bioactive Compounds	MIC *	Organism Inhibited	References
*Allium sativum* L.	Alliaceae	Methanol	Cyanidin-3-(6’-malonyl)-glucoside, vanillic acid caffeic acid, p-coumaric acid, ferulic acid, sinapic acid, L-alliin, alliin isomer and methiin	-	*B. cereus, L. monocytogenes S. aureus, P. aeruginosa, E. coli*	[11]
*Searsia chirindensis* (Baker f.) Moffett	Anacardiaceae	Ethanol	Methyl gallate	30–130 μg/mL	*C. jejuni, E. coli, S. flexneri, S. aureus*	[86]
			myricetin-3-O-arabinopyranoside	60–250 μg/mL		
			myricetrin-3-O-rhamnoside	60–250 μg/mL		
			kaempferol-3-O-rhamnoside	130–250 μg/mL		
			quercetin-3-O-arabinofuranoside	250 μg/mL		
		Dichloromethane/Methanol		250–6250 μg/mL		
		n-butanol		130–3125 μg/mL		
		Ethyl Acetate		60–780 μg/mL		
		Crude		60–780 μg/mL		
*Xylopia aethiopica* (Dunal) A. Rich.	Annonaceae	Aqueous	1R-a-Pinene, β-Pinene, 2-Carene, Cyclohexene,5-methyl-3-(1-methylethenyl)-trans-(-)- Bicyclo [3.1.0] hexane,6-isopropylidene-1-methyl-, Eucalyptol, Ethyl 2-(5-methyl-5-vinyltetrahydrofuran-2-yl) propan-2-yl carbonate, Isogeraniol, ɑ-Campholenal, L-trans-Pinocarveol, Pinocarvone, Myrtenal, (-)-Spathulenol	1–256 μg/mL	*S. aureus, B. licheniformis, E. coli, K. pneumoniae*	[87]
*Polyalthia cerasoides*	Annonaceae	Hexane	N-(4-hydroxy-β-phenethyl-4-hydroxy cinnamide	64–128 μg/mL	*C. diphtheria, B. subtilis, B. cereus, M. lutens*	[88]
		Dichloromethane		32–256 μg/mL		
*Unonopsis lindmanii* R. E. Fries	Anonaceae	Hexane	Gallic acid, kaempferol, ellagic acid, epicatechin, vitexin, corilagin	25–250 μg/mL	*C.albicans*	[89]
*Allagoptera leucocalyx* (Drude) Kuntze,	Arecaceae	Hexane	Gallic acid, kaempferol, ellagic acid, epicatechin, vitexin, corilagin	162.2–665 mg/mL	*C.albicans*	[89]
*Bactris glaucescens* Drude	Arecaceae	Hexane	Gallic acid, kaempferol, ellagic acid, epicatechin, vitexin, corilagin	200–755 mg/mL	*C.albicans*	[89]
*Scheelea phalerata* Mart	Arecaceae	Hexane	Gallic acid, kaempferol, ellagic acid, epicatechin, vitexin, corilagin	129 mg/mL	*C.albicans*	[89]
*Artemisia herba-alba* Asso	Asteraceae	Aqueous	1,8-cineole, β-thujone, α-thujone, camphor	640–2500 μg/mL	*T. rubrum and E. floccosum*	[90]
*Vernonia adoensis* Sch. Bip. ex Walp.	Asteraceae	Acetone	Chondrillasterol	50 μg/mL	*S. aureus, K. pneumonia, P. aeruginosa*	[1]
*Matricaria chamomilla*	Asteraceae	Ethanol	Phenolic acid	1.56–3.12 mg/mL	*S. typhimurium*	[19]
*Solidago graminifolia* L. Salisb.	Asteraceae	Ethanol	di-C-glycosylflavones (schaftoside, isoschaftoside), caftaric acid, gentisic acid, chlorogenic acid, *p*-coumaric acid, ferulic acid, hyperoside, rutin, quercitrin, quercetin, Luteolin, kaempferol, gallic acid, protocatechuic acid, vanillic acid, syringic acid, rosmarinic acid	40–3120 μg/mL	*S. aureus, C. albicans, C. parapsilosis*.	[12]
		Methanol		90–3120 μg/mL		
		Aqueous		190–6250 μg/mL		
*Baccharis trimera*	Asteraceae	Crude	Polyphenols, flavonoids, alkaloids, and terpenes	7.8–500 μg/mL	*E. coli, S. aureus, P. aeruginosa, C. albicans, C. tropicalis, C. parapsilosis, Epicoccum sp., C. sphaerospermum, C. neoformans, P. brasiliensis, C. gatti, Pestalotiopsis sp., C. lunatus, Nigrospora sp.*	[88]
*Tecoma stans*	Bignoniaceae	Aqueous	Phenolic compounds	50–600 μg/mL	*S. aureus*	[91]
*Bixa orellana* L.	Bixaceae	Aqueous	Bixin, catechin, chlorogenic acid, chrysin, butein, hypolaetin, licochalcone A, and xanthohumol.	16–32 μg/mL	*B. cereus, S. aureus*	[9]
*Trichodesma indicum*	Boraginaceae	Ethanol	Lanast-5-en-3β-D- glucopyranosyl-21(24)-oilde	2.4–19.2 μg/mL	*S. aureus*	[92]
*Boswellia dalzielii* Hutch.	Burseraceae	Crude	Oleic acid, squalene and n-hexadecanoic acid	-	*S. pyogenes*, *S. aureus*, *E. coli*, *E. faecalis*, *K. pneumonia*, *P. aeruginosa*, *P. mirabilis*, *S. typhi*, and *C. albicans*	[93]
*Caesalpinia coriaria* (Jacq) Willd	Caesalpiniaceae	Aqueous	Methyl gallate and gallic acid	1.56–25 mg/mL	S. typhi, E. coli, P. aeruginosa, L. monocytogenes, S. aureus.	[94]
		Ethanol		390–6250 μg/mL		
*Senna aculeate* (Bth.) Irw et Barn	Ceasalpinioideae	Hexane	Gallic acid, kaempferol, ellagic acid, epicatechin, vitexin, corilagin	25, 50, 100 mg/mL	*C.albicans*	[89]
*Kochia scoparia*	Chenopodiaceae	Crude	Polyphenols, flavonoids, alkaloids, and terpenes	3.125 mg/mL	*C. graminicola, T. deformans, A. flavus, H. carbonum, C. zeaemaydis, C. macrocarpum, P. innundatus, S. japonicas, E. ficariae, P. herbarum, M. verticillata, Rhisoclosmatium* sp., *S. pseudodichotomus, S. kneipii, R. solani, P. sojae.*	[8]
*Buchenavia tomentosa* (Mart) Eichler	Combretaceae	Hexane	Gallic acid, Kaempferol, Ellagic acid, epicatechin, Vitexin, Corilagin	10 mg/mL	*C.albicans*	[89]
*Terminalia phanerophlebia* Engl. & Diels	Combretaceae	Crude	Methyl gallate (methyl-3,4,5-trihydroxybenzoate) and a phenylpropanoid glucoside, 1,6-di-O-coumaroyl glucopyranoside	125 μg/mL	*M. aurum, M. tuberculosis, S. aureus, K. pneumoniae*	[95]
		Dichloromethane		16–250 μg/mL		
		Hexane		31–250 μg/mL		
		Ethyl Acetate		8–125 μg/mL		
		n-butanol		31–250 μg/mL		
*Buchenavia tomentosa* L.	Combretaceae	Crude	Gallic acid, quinic acid, kaempferol, (-) epicatechin, ellagic acid, buchenavianine, eschweilenol b, eschweilenol c, vitexin, corilagin, 1α,23β-dihydroxy-12-oleanen-29-oicacid-23β-o-α-l-4-acetylramnopiranoside and punicalin	200–12500 μg/mL	*Candida albicans, Candida tropicalis, Candida parapsilosis, Candida glabrata, Candida krusei and Candida dubliniensis.*	[96]
*Diadema setosum f. depressa* Dollfus & Roman.	Diadematidae	Acetone	Polyunsaturated fatty acids (PUFAs) and β-carotene	500–4000 μg/mL	*S. typhi, S. typhimurium, S. flexneri, P. aeruginosa, A. hydrophila, Acinetobacter sp, C. freundii and K. pneumonia, B. subtilis, S. epidermidis S. aureus*	[1]
*Monotes kerstingii* Gilg	Dipterocarpaceae	Crude	Stilbene-coumarin derivative, coumarin-carbinol and fatty glycoside	1–8 mg/mL	*B. subtilis, Septoria tritici* Desm	[7]
*Croton doctoris* S Moore	Euphorbiaceae	Hexane	Gallic acid, kaempferol, ellagic acid, epicatechin, vitexin, corilagin	500 μg/mL	*C.albicans*	[89]
*Jatropha weddelliana* Baillon	Euphorbiaceae	Hexane	Gallic acid, kaempferol, ellagic acid, epicatechin, vitexin, corilagin	4–32 μg/mL	*C.albicans*	[89]
*Cassia alata*	Fabaceae	Ethanol	4-butylamine, cannabinoid, dronabinol, methyl-6-hydroxy	1.25, 1.5 mg/mL	*S. aureus, E. coli, P. aeruginosa, C. albicans*	[28]
*Dalbergia scandens* Roxb., Corom.	Fabaceae	Ethanol	Dalpanitin, vicenin-2 and 3, rutin	780–6250 mg/mL	*B. cereus, S. aureus, E. coli, P. aeruginosa, C. albicans*	[41]
*Acacia nilotica*	Fabaceae	Crude	Alkaloids	600–1200 μg/mL	*S. aureus*	[27]
*Salvia sessei Benth*	Lamiaceae	Hexane	Sessein, isosessein	12.5–100 μg/mL	*S. haemolyticus, S. hominis, E. faecalis, S. epidermis, S. pyogenes, S.aureus*	[14]
		Dichloromethane		100 μg/mL		
		Methanol		12.5–100 μg/mL		
*Mentha piperita*	Lamiaceae	Methanol	1,1-diphenyl-2-picrylhydazyl-hydrate	1–4 mg/mL	*S. aureus, E. coli, C. albicans*	[97]
*Ocimum basilicum* L.	Lamiaceae	Ethanol	Gallic acid, 3,4-dihydroxy benzoic acid, 4-hydroxy benzoic acid, 2,5 dihydroxybenzoic acid, chlorogenic acid, vanillic acid, Epicatechin, caffeic acid, p-coumaric acid, ferulic acid, rutin, ellagic acid, naringin, quercetin, cinnamic acid, α-pinene, camphene, sabinene, β-pinene, myrcene, 3-octanol, α-terpinene, *p*-cymene, limonene, 1,8-cineole, *(Z)*-β-ocimene, *(E)*-β-ocimene, γ-terpinene, *cis*-sabinene hydrate, terpinolene, linalool, nonanal, pentylisovalerate, 1-octen-3-yl acetate, *cis-p*-menth-2-en-1-ol, 3-octyl acetate, α-campholenal, camphor, *trans*-verbenol, δ-terpineol, 4-terpineol, α-terpineol, *cis*-dihydrocarvone, *trans*-carveol, *(Z)*-3-hexenyl isovalerate, pulegone, neral, carvone, linalyl acetate, bornyl acetate, dihydroedulan IA, isodihydrocarvyl acetate, α-terpinyl acetate, *cis*-carvyl acetate, neryl acetate, geranyl acetate, β-elemene, *(Z)*-jasmone, β-caryophyllene, β-copaene, aromadendrene, α-humulene, *(E)*-β-farnesene, *cis*-muurola-4(14), 5-diene germacrene D, bicyclogermacrene, germacrene A, δ-cadinene, *(E)*-α-bisabolene, *(E)*-nerolidol, Spathulenol, caryophyllene oxide, viridiflorol, 1, 10-di-*epi*-cubenol, T-cadinol, T-muurolol, monoterpene hydrocarbons, oxygenated monoterpenes, sesquiterpene hydrocarbons, oxygenated sesquiterpenes, apocarotenes non-terpene derivatives	16–256 μg/mL	*S. epidermidis S. aureus, B. subtilis*, *E. coli*, *P. aeruginosa*, *K. pneumoniae*, *C. glabrata, C. albicans*	[98]
*Thymus algeriensis* Boiss. & Reut	Lamiaceae	Ethanol	Gallic acid, 3,4-dihydroxy benzoic acid, 4-hydroxy benzoic acid, 2,5 dihydroxybenzoic acid, chlorogenic acid, vanillic acid, epicatechin, caffeic acid, p-coumaric acid, ferulic acid, rutin, ellagic acid, naringin, quercetin, cinnamic acid, α-pinene, camphene, sabinene, β-pinene, myrcene, 3-octanol, α-terpinene, *p*-cymene, limonene, 1,8-cineole, *(Z)*-β-ocimene, *(E)*-β-ocimene, γ-terpinene, *cis*-sabinene hydrate, terpinolene, linalool, nonanal, pentylisovalerate, 1-octen-3-yl acetate, *cis-p*-menth-2-en-1-ol, 3-octyl acetate, α-campholenal, camphor, *trans*-verbenol, δ-terpineol, 4-terpineol, α-terpineol, *cis*-dihydrocarvone, *trans*-carveol, *(Z)*-3-hexenyl isovalerate, pulegone, neral, carvone, linalyl acetate, bornyl acetate, dihydroedulan IA, isodihydrocarvyl acetate, α-terpinyl acetate, *cis*-carvyl acetate, neryl acetate, geranyl acetate, β-elemene, *(Z)*-jasmone, β-caryophyllene, β-copaene, aromadendrene, α-humulene, *(E)*-β-farnesene, *cis*-muurola-4(14), 5-diene germacrene D, bicyclogermacrene, germacrene A, δ-cadinene, *(E)*-α-bisabolene, *(E)*-nerolidol, spathulenol, caryophyllene oxide, viridiflorol, 1, 10-di-*epi*-cubenol, T-cadinol, T-muurolol, monoterpene hydrocarbons, oxygenated monoterpenes, sesquiterpene hydrocarbons, oxygenated sesquiterpenes, apocarotenes non-terpene derivatives	32–512 μg/mL	*S. epidermidis S. aureus, B. subtilis*, *E. coli*, *P. aeruginosa*, *K. pneumoniae*, *C. glabrata, C. albicans*	[98]
*Cinnamomun inerme*	Lauraceae	Ethyl Acetate	5-(1,5-dimethyl-2-4-hexenyl)- methyl phenol)	100–800 μg/mL	*S. aureus, E. coli*	[99]
		Hexane		8000 μg/mL		
		Acetone		8000 μg/mL		
		n-butanol		100–800 μg/mL		
*Allium sativam*	Liliaceae	Crude	Allicin	49 μg/mL	*C. albicans*	[100]
*Strychnos nigritana* Baker	Loganiaceae	Crude	Nigritanine, Speciociliatine, Mytragine Paynantheine Rhyncophylline	128–256 μg/mL	S. aureus	[10]
*Mascagnia benthamiana* (Gries) WR Anderson	Malpighiaceae	Hexane	Gallic acid, kaempferol, ellagic acid, epicatechin, vitexin, corilagin	17.84 mg/mL	*C.albicans*	[89]
*Mouriri elliptica* Mart	Memecylaceae	Hexane	Gallic acid, kaempferol, ellagic acid, epicatechin, vitexin, corilagin	100 μg/mL	*C.albicans*	[89]
*Artocarpus communis*	Moraceae	Crude	Atonin E, 2-(3,5-dihydroxy)-(Z)-4-(3 methyl but-1-etnyl	4–512 μg/mL	*P. aeruginosa, S.typhi, S.aureus, K.pneumoniae*	[101]
Myrtus nivellei Batt. & Trab.	Myrtaceae	Crude	1,8-cineole, limonene, isoamylcyclopentane, di-nor-sesquiterpenoids	5 mg/mL	*C. neoformans*	[102]
Myrtus communis L	Myrtaceae	Crude	α-pinene, 1,8-cineole, linalool, and linalyl acetate	156–625 μg/mL	*E. floccosum, M. canis, T. rubrum*	[102]
*Piper nigrum*	Piperaceae	Aqueous	Piperine	500–1000 μg/mL	*E. coli, M. luteus*	[91]
*Citrus aurantium* L.	Rutaceae	Ethanol	Polyphenols, flavonoids, alkaloids, and terpenes	1562–6250 μg/mL	Amoxycillin resistant *B. cereus*	[13]
*Salix babylonica* L.	Salicaceae	Hydroalcoholic	Luteolin, luteolin 7-*O*-glucoside	1.56–100 mg/mL	*E. coli, S. aureus* and *L. monocytogenes*	[103]
*Verbascum glabratum* subsp. bosnense (K. Malý) Murb	Scrophulariaceae	Ethanol	quercitrin and rosmarinic acid, 4-hydroxybenzoic acid, salicylic acid, morin, and apigenin	600, 1200 μg/mL	*E. coli, S. aureus, Candida albicans*	[17]
*Simaba ferruginea* A. St.-Hil	Simaroubaceae	Methanol	Canthin-6-one, indole β-carboxylic	12.5–200 μg/mL	S. flexneri, S. aureus and S. aureus	[91]
*Camellia sinensis*	Theaceae	Aqueous	Catechin	7.81–31.25 μg/mL	*S. mutans*	[104]
Talaromyces sp.	Trichocomaceae	Aqueous	Talaropeptide A and B	5 mg/mL	*B. subtilis*	[18]
*Hybanthus enneasperm* *us*	Violaceae	Crude	Flavonoids, Tannins	37.5, 75, 150, 300, 600 μg/mL	*P. vulgaris, V. cholera*	[100]

* MIC (minimum inhibitory concentration) is the lowest drug concentration at which a given antimicrobial extract inhibits the visible growth of a tested organism. **MIC absolute value**: the given absolute value of drug concentration inhibits the growth of all tested organisms/**MIC ranges**: the given range of drug concentrations (minimum to maximum) inhibit the growth of the individual to all tested organisms.

## Abbreviations

*A. bohemicus*	*Acinetobacter bohemicus*
*A. flavus*	*Aspergillus flavus*
*A. fumigatus*	*Aspergillus fumigatus*
*A. niger*	*Aspergillus niger*
*A. solani*	*Alternaria solani*
*B. agri*	*Brevibacillus agri*
*B. brevis*	*Brevibacillus brevis*
*B. cereus*	*Bacillus cereus*
*B. megaterium*	*Bacillus megaterium*
*B. pumilus*	*Bacillus pumilus*
*B. subtilis*	*Bacillus subtilis*
*C. albicans*	*Candida albicans*
*C. Dipthieriae*	*Corynebacterium Dipthieriae*
*C. dubliniensis*	*Candida dubliniensis*
*C. glabrata*	*Candida glabrata*
*C. graminicola*	*Colletotrichum graminicola*
*C. jejuni*	*Campylobacter jejuni*
*C. krusei*	*Candida krusei*
*C. lunat*	*Candida lunat*
*C. lunatus*	*Cochliobolus lunatus*
*C. macrocarpum*	*Cladosporium macrocarpum*
*C. neoformans*	*Cryptococcus neoformans*
*C. parapsilosis*	*Candida parapsilosis*
*C. sphaerospermum*	*Cladosporium sphaerospermum*
*C. tropicalis*	*Candida tropicalis*
*C. maydis*	*Cercospora zeae-maydis*
*D. turcica*	*Drechslera turcica*
*E. aerogenes*	*Enterobacter aerogenes*
*E. cloacae*	*Enterobacter cloacae*
*E. coli*	*Escherichia coli*
*E. faecalis*	*Enterococcus faecalis*
*E. ficariae*	*Entyloma ficariae*
*E. floccosum*	*Epidermophyton floccosum*
*F. nucleatum*	*Fusobacterium nucleatum*
*F. oxysporum*	*Fusarium oxysporum*
*F. verticillioides*	*Fusarium verticillioides*
*H. carbonum*	*Helminthosporium carbonum*
*H. pylori*	*Helicobacter pylori*
*K. aerogenes*	*Klebsiella aerogenes*
*K. kristinae*	*Kocuria kristinae*
*K. pneumonia*	*Klebsiella pneumonia*
*L. acidophilus*	*Lactobacillus acidophilus*
*L. casei*	*Lactobacillus casei*
*L. innocua*	*Listeria innocua*
*L. monocytogenes*	*Listeria monocytogenes*
*L. sporogenes*	*Lactobacillus sporogenes*
*M. canis*	*Microsporum canis*
*M. luteus*	*Micrococcus luteus*
*M. morganii*	*Morganella morganii*
*M. ruber*	*Monascus ruber*
*M. smegmatis*	*Mycobacterium smegmatis*
*M. tuberculosis*	*Mycobacterium tuberculosis*
*M. verticillata*	*Mortierella verticillata*
*P. acnes*	*Propionibacterium acnes*
*P. aeruginosa*	*Pseudomonas aeruginosa*
*P. brasiliensis*	*Paracoccidioides brasiliensis*
*P. fluorescens*	*Pseudomonas fluorescens*
*P. gingivalis*	*Porphrymonas gingivalis*
*P. herbarum*	*Pleospora herbarum*
*P. innundatus*	*Protomyces innundatus*
*P. intermedia*	*Prevotella intermedia*
*P. lilacinum*	*Purpureocillium lilacinum*
*P. mirabilis*	*Proteus mirabilis*
*P. sojae*	*Phytophthora sojae*
*P. vulgaris*	*Proteus vulgaris*
*R. rubrum*	*Rhodospirillum rubrum*
*R. solanacearum*	*Ralstonia solanacearum*
*R. solani*	*Rhizoctonia solani*
*R. stolonifera*	*Rhizopus stolonifera*
*S. agalactiae*	*Streptococcus agalactiae*
*S. anginosus*	*Streptococcus anginosus*
*S. aureus*	*Staphylococcus aureus*
*S. auricularis*	*Staphylococcus auricularis*
*S. boydii*	*Shigella boydii*
*S. dysenteriae*	*shigella dysenteriae*
*S. epidermidis*	*Staphylococcus epidermidis*
*S. fecalis*	*Streptococcus fecalis*
*S. flexneri*	*Shigella flexneri*
*S. gordonii*	*Streptococcus gordonii*
*S. haemolyticus*	*Staphylococcus haemolyticus*
*S. heidelberg*	*Salmonella heidelberg*
*S. hominis*	*Staphylococcus hominis*
*S. japonicas*	*Schizosaccharomyces japonicas*
*S. kneipii*	*Spizellomyces kneipii*
*S. lutea*	*Sarcina lutea*
*S. marcescens*	*Serratia marcescens*
*S. mutans*	*Streptococcus mutans*
*S. para typhi*	*Salmonella para typhi*
*S. pneumoniae*	*Streptococcus pneumoniae*
*S. pseudodichotomus*	*Spizellomyces pseudodichotomus*
*S. pyogenes*	*Streptococcus pyogenes*
*S. sanguis*	*Streptococcus sanguis*
*S. saprophyticus*	*Staphylococcus saprophyticus*
*S. shiga*	*Shigella shiga*
*S. typhi*	*Salmonella typhi*
*T. deformans*	*Taphrina deformans*
*T. mentagraphytes*	*Trichophyton mentagraphytes*
*T. rubrum*	*Trichophyton rubrum*
*T. tonsurans*	*Trichophyton tonsurans*
*T. urans*	*Trichophytontonsurans*
*V. cholerae*	*Vibrio cholerae*
*V. fischeri*	*Vibrio fischeri*
*X. axonopodis Pv. malvacearum*	*Xanthomonas axonopodis pv. Malvacearum*
*X. vesicatoria*	*Xanthomonas vesicatoria*
*Y. enterocolitica*	*Yersinia enterocolitica*

## References

[1] Mozirandi W., Tagwireyi D., Mukanganyama S. (2019). Evaluation of antimicrobial activity of chondrillasterol isolated from Vernonia adoensis (Asteraceae). BMC Complement. Altern. Med..

[2] Mickymaray S. (2019). One-step synthesis of silver nanoparticles using Saudi Arabian desert seasonal plant *Sisymbrium irio* and antibacterial activity against multidrug-resistant bacterial strains. Biomolecules.

[3] Kannaiyan M., Manuel V.N., Raja V., Thambidurai P., Mickymaray S., Nooruddin T. (2012). Antimicrobial activity of the ethanolic and aqueous extracts of Salacia chinensis Linn. against human pathogens. Asian Pac. J. Trop. Dis..

[4] Kannaiyan M., Meseret Abebe G., Kanimozhi C., Thambidurai P., Ashokapuram Selvam S., Vinodhini R., Suresh M. (2018). Prevalence of extended-spectrum beta-lactamase producing enterobacteriaceae members isolated from clinically suspected patients. Asian J. Pharm. Clin. Res..

[5] Vijayakumar R., Aboody M., AlFonaisan M., Turaiki W., Mickymaray S., Mariappan P., Alsagaby S., Sandle T. (2016). Determination of Minimum inhibitory concentrations of Common Biocides to Multidrug-Resistant Gram-negative bacteria. Appl. Med. Res..

[6] Mickymaray S., Alturaiki W. (2018). Antifungal efficacy of marine macroalgae against fungal isolates from bronchial asthmatic cases. Molecules.

[7] Fotso G.W., Mogue Kamdem L., Dube M., Fobofou S.A., Ndjie Ebene A., Arnold N., Tchaleu Ngadjui B. (2019). Antimicrobial secondary metabolites from the stem barks and leaves of Monotes kerstingii Gilg (Dipterocarpaceae). Fitoterapia.

[8] Houlihan A.J., Conlin P., Chee-Sanford J.C. (2019). Water-soluble exudates from seeds of Kochia scoparia exhibit antifungal activity against Colletotrichum graminicola. PLoS ONE.

[9] Mickymaray S., Al Aboody M.S., Rath P.K., Annamalai P., Nooruddin T. (2016). Screening and antibacterial efficacy of selected Indian medicinal plants. Asian Pac. J. Trop. Biomed..

[10] Casciaro B., Calcaterra A., Cappiello F., Mori M., Loffredo M.R., Ghirga F., Mangoni M.L., Botta B., Quaglio D. (2019). Nigritanine as a New Potential Antimicrobial Alkaloid for the Treatment of Staphylococcus aureus-Induced Infections. Toxins.

[11] Phan A.D.T., Netzel G., Chhim P., Netzel M.E., Sultanbawa Y. (2019). Phytochemical Characteristics and Antimicrobial Activity of Australian Grown Garlic (*Allium Sativum* L.) Cultivars. Foods.

[12] Toiu A., Vlase L., Vodnar D.C., Gheldiu A.-M., Oniga I. (2019). *Solidago graminifolia* L. Salisb. (Asteraceae) as a Valuable Source of Bioactive Polyphenols: HPLC Profile, In Vitro Antioxidant and Antimicrobial Potential. Molecules.

[13] Değirmenci H., Erkurt H. (2019). Relationship between volatile components, antimicrobial and antioxidant properties of the essential oil, hydrosol and extracts of *Citrus aurantium* L. flowers. J. Infect. Public Health.

[14] Gómez-Rivera A., González-Cortazar M., Herrera-Ruíz M., Zamilpa A., Rodríguez-López V. (2018). Sessein and isosessein with anti-inflammatory, antibacterial and antioxidant activity isolated from Salvia sessei Benth. J. Ethnopharmacol..

[15] Sukalingam K., Ganesan K., Ponnusamy K. (2015). Evaluation of antidiabetic activity of polyherbal formulations on type 2 diabetic patients: A single blinded randomized study. Int. J. Integ. Medl. Sci..

[16] Sukalingam K., Ganesan K., Xu B. (2017). *Trianthema portulacastrum* L. (giant pigweed): Phytochemistry and pharmacological properties. Phytochem. Rev..

[17] Karalija E., Parić A., Dahija S., Bešta-Gajević R., Ćavar Zeljković S. (2018). Phenolic compounds and bioactive properties of Verbascum glabratum subsp. bosnense (K. Malý) Murb., an endemic plant species. Nat. Prod. Res..

[18] Dewapriya P., Khalil Z.G., Prasad P., Salim A.A., Cruz-Morales P., Marcellin E., Capon R.J. (2018). Talaropeptides A-D: Structure and Biosynthesis of Extensively N-methylated Linear Peptides From an Australian Marine Tunicate-Derived Talaromyces sp.. Front. Chem..

[19] Nath D., Banerjee P., Shaw M., Mukhopadhyay M.K. (2018). Bottle Gourd (Lagenaria Siceraria). Fruit and Vegetable Phytochemicals: Chemistry and Human Health.

[20] Prasannabalaji N., Muralitharan G., Sivanandan R.N., Kumaran S., Pugazhvendan S.R. (2012). Antibacterial activities of some Indian traditional plant extracts. Asian Pac. J. Trop. Dis..

[21] Mabona U., Viljoen A., Shikanga E., Marston A., Van Vuuren S. (2013). Antimicrobial activity of southern African medicinal plants with dermatological relevance: From an ethnopharmacological screening approach, to combination studies and the isolation of a bioactive compound. J. Ethnopharmacol..

[22] Benevides Bahiense J., Marques F.M., Figueira M.M., Vargas T.S., Kondratyuk T.P., Endringer D.C., Scherer R., Fronza M. (2017). Potential anti-inflammatory, antioxidant and antimicrobial activities ofSambucus australis. Pharm. Biol..

[23] Akhalwaya S., van Vuuren S., Patel M. (2018). An in vitro investigation of indigenous South African medicinal plants used to treat oral infections. J. Ethnopharmacol..

[24] Lim S.S., Selvaraj A., Ng Z.Y., Palanisamy M., Mickmaray S., Cheong P.C.H., Lim R.L.H. (2018). Isolation of actinomycetes with antibacterial activity against multi-drug resistant bacteria. Malays. J. Microbiol..

[25] Ke Y., Al Aboody M.S., Alturaiki W., Alsagaby S.A., Alfaiz F.A., Veeraraghavan V.P., Mickymaray S. (2019). Photosynthesized gold nanoparticles from Catharanthus roseus induces caspase-mediated apoptosis in cervical cancer cells (HeLa). Artif. Cells Nanomed. Biotechnol..

[26] Muhaisen H.M.H., Ab–Mous M.M., Ddeeb F.A., Rtemi A.A., Taba O.M., Parveen M. (2015). Antimicrobial agents from selected medicinal plants in Libya. Chin. J. Integr. Med..

[27] Mubarack H., Doss A., Vijayasanthi M., Venkataswamy R. (2012). Antimicrobial drug susceptibility of Staphylococcus aureus from subclinical bovine mastitis in Coimbatore, Tamilnadu, South India. Vet. World.

[28] Okwu M.U., Olley M., Akpoka A.O., Izevbuwa O.E. (2019). Methicillin-resistant Staphylococcus aureus (MRSA) and anti-MRSA activities of extracts of some medicinal plants: A brief review. AIMS Microbiol..

[29] Obeidat M., Shatnawi M., Al-alawi M., Al-Zu‘bi E., Al-Dmoor H., Al-Qudah M., El-Qudah J., Otri I. (2012). Antimicrobial Activity of Crude Extracts of Some Plant Leaves. Res. J. Microbiol..

[30] (2016). Aristolochia indica Linn. SpringerReference.

[31] Vinodhini R., Moorthy K., Suresh M. (2016). Incidence and virulence traits of *Candida dubliniensis* isolated from clinically suspected patients. Asian J. Pharm. Clin. Res..

[32] Zuo G.-Y., Zhang X.-J., Yang C.-X., Han J., Wang G.-C., Bian Z.-Q. (2012). Evaluation of Traditional Chinese Medicinal Plants for Anti-MRSA Activity with Reference to the Treatment Record of Infectious Diseases. Molecules.

[33] Singh A., Bajpai V., Kumar S., Kumar B., Srivastava M., Arya K.R., Sharma K.R. (2016). Distribution and Discrimination Study of Bioactive Compounds from Berberis species using HPLC-ESI-QTOF-MS/MS with Principle Component Analysis. Nat. Prod. Commun..

[34] Kariu T., Nakao R., Ikeda T., Nakashima K., Potempa J., Imamura T. (2016). Inhibition of gingipains andPorphyromonas gingivalisgrowth and biofilm formation by prenyl flavonoids. J. Periodontal Res..

[35] (2016). Spathodea campanulata Beauv. SpringerReference.

[36] dos Santos E., Pereira M., da Silva C., Souza-Neta L., Geris R., Martins D., Santana A., Barbosa L., Silva H., Freitas G. (2013). Antibacterial Activity of the Alkaloid-Enriched Extract from Prosopis juliflora Pods and Its Influence on in Vitro Ruminal Digestion. Int. J. Mol. Sci..

[37] Kumar G., Maheswaran R., Sharmila Banu G. (2013). Antihyperlipideamic effect of *Solanum trilobatum* L. leaves extract on streptozotocin induced diabetic rats. Asian J. Biomed. Pharm. Sci..

[38] Semalty M., Semalty A., Badola A., Joshi G., Rawat M.S.M. (2010). Semecarpus anacardium Linn.: A review. Pharm. Rev..

[39] Rawat S., Jugran A.K., Bahukhandi A., Bahuguna A., Bhatt I.D., Rawal R.S., Dhar U. (2016). Anti-oxidant and anti-microbial properties of some ethno-therapeutically important medicinal plants of Indian Himalayan Region. 3 Biotech..

[40] Gujjeti R.P., Namthabad S., Mamidala E. (2014). HIV-1 reverse transcriptase inhibitory activity of Aerva lanata plant extracts. BMC Infect. Dis..

[41] Mohotti S., Rajendran S., Muhammad T., Strömstedt A.A., Adhikari A., Burman R., de Silva E.D., Göransson U., Hettiarachchi C.M., Gunasekera S. (2020). Screening for bioactive secondary metabolites in Sri Lankan medicinal plants by microfractionation and targeted isolation of antimicrobial flavonoids from Derris scandens. J. Ethnopharmacol..

[42] Ghasemi P., Jahanbazi P., Enteshari S., Malekpoor F., Hamedi B. (2010). Antimicrobial activity of some Iranian medicinal plants. Arch. Biol. Sci..

[43] Mickymaray S., Al Aboody M.S. (2019). In Vitro Antioxidant and Bactericidal Efficacy of 15 Common Spices: Novel Therapeutics for Urinary Tract Infections?. Medicina.

[44] Banothu V., Neelagiri C., Adepally U., Lingam J., Bommareddy K. (2017). Phytochemical screening and evaluation of in vitro antioxidant and antimicrobial activities of the indigenous medicinal plant Albizia odoratissima. Pharm. Biol..

[45] Sahu M.C., Padhy R.N. (2013). In vitro antibacterial potency of Butea monosperma Lam. against 12 clinically isolated multidrug resistant bacteria. Asian Pac. J. Trop. Dis..

[46] Wikaningtyas P., Sukandar E.Y. (2016). The antibacterial activity of selected plants towards resistant bacteria isolated from clinical specimens. Asian Pac. J. Trop. Biomed..

[47] Mwinga J.L., Asong J.A., Amoo S.O., Nkadimeng S.M., McGaw L.J., Aremu A.O., Otang-Mbeng W. (2019). In vitro antimicrobial effects of Hypoxis hemerocallidea against six pathogens with dermatological relevance and its phytochemical characterization and cytotoxicity evaluation. J. Ethnopharmacol..

[48] Armas J., Quiroz J., Roman R., Sanchez J., Pacheco M., Valdivia L., Rivera E., Asmat R., Anampa A. (2016). Antibacterial Activities of Essential Oils from Three Medicinal Plants in Combination with EDTA against Methicillin-resistant Staphylococcus aureus. Br. Microbiol. Res. J..

[49] Ferhat M., Erol E., Beladjila K.A., Çetintaş Y., Duru M.E., Öztürk M., Kabouche A., Kabouche Z. (2016). Antioxidant, anticholinesterase and antibacterial activities of Stachys guyoniana and Mentha aquatica. Pharm. Biol..

[50] Guan C.P., Luo H.X., Fang H.E., Zhou X.Z. (2018). Global Transcriptome Changes of Biofilm-Forming Staphylococcus epidermidis Responding to Total Alkaloids of Sophorea alopecuroides. Pol. J. Microbiol..

[51] Zhou J.-X., Braun M., Wetterauer P., Wetterauer B., Wink M. (2019). Antioxidant, Cytotoxic, and Antimicrobial Activities of Glycyrrhiza glabra L., Paeonia lactiflora Pall., and Eriobotrya japonica (Thunb.) Lindl. Extracts. Medicines.

[52] Arefin M.K., Rahman M.M., Uddin M.Z., Hassan M.A. (1970). Angiosperm flora of Satchari National Park, Habiganj, Bangladesh. Bangladesh J. Plant. Taxon..

[53] Koona S., Budida S. (2011). Antibacterial Potential of the Extracts of the Leaves of Azadirachta indica Linn. Not. Sci. Biol..

[54] Durairaj B., Dorai A. (2010). Antiplatelet activity of white and pink Nelumbo nucifera Gaertn flowers. Braz. J. Pharm. Sci..

[55] Bhattacharjee I., Chatterjee S.K., Chandra G. (2010). Isolation and identification of antibacterial components in seed extracts of Argemone mexicana L. (Papaveraceae). Asian Pac. J. Trop. Med..

[56] Jayachandran M., Zhang T., Ganesan K., Xu B., Chung S.S.M. (2018). Isoquercetin ameliorates hyperglycemia and regulates key enzymes of glucose metabolism via insulin signaling pathway in streptozotocin-induced diabetic rats. Eur. J. Pharmcol..

[57] Chatterjee S.K., Bhattacharjee I., Chandra G. (2011). Isolation and identification of bioactive antibacterial components in leaf extracts of Vangueria spinosa (Rubiaceae). Asian Pac. J. Trop. Med..

[58] Gaziano R., Campione E., Iacovelli F., Marino D., Pica F., Di Francesco P., Aquaro S., Menichini F., Falconi M., Bianchi L. (2018). Antifungal activity of Cardiospermum halicacabum L. (Sapindaceae) against Trichophyton rubrum occurs through molecular interaction with fungal Hsp90. Drug Des. Dev..

[59] Nefzi A., Ben Abdallah R.A. (2016). Antifungal activity of aqueous and organic extracts from Withania somnifera L. against Fusarium oxysporum f. sp. Radicis lycopersici. J. Microb. Biochem. Technol..

[60] Chahal S.S., Matthews H.R., Bradbury E.M. (1980). Acetylation of histone H4 and its role in chromatin structure and function. Nature.

[61] Venkataswamy R., Doss A., Sukumar M., Mubarack H.M. (2010). Preliminary phytochemical screening and antimicrobial studies of Lantana indica roxb. Ind. J. Pharm. Sci..

[62] Pandian M.R., Banu G.S., Kumar G. (2006). A study of the antimicrobial activity of Alangium salviifolium. Indian J. Pharm..

[63] Arulmozhi P., Vijayakumar S., Kumar T. (2018). Phytochemical analysis and antimicrobial activity of some medicinal plants against selected pathogenic microorganisms. Microb. Pathog..

[64] Kahaliw W., Aseffa A., Abebe M., Teferi M., Engidawork E. (2017). Evaluation of the antimycobacterial activity of crude extracts and solvent fractions of selected Ethiopian medicinal plants. BMC Complement. Altern. Med..

[65] Ginovyan M., Petrosyan M., Trchounian A. (2017). Antimicrobial activity of some plant materials used in Armenian traditional medicine. BMC Complement. Altern. Med..

[66] Asgarpanah J., Hashemi S.J., Hashemi E., Askari K. (2015). In vitro antifungal activity of some traditional Persian medicinal plants on pathogenic fungi. Chin. J. Integ. Med..

[67] Sharma A., Flores-Vallejo R.D.C., Cardoso-Taketa A., Villarreal M.L. (2017). Antibacterial activities of medicinal plants used in Mexican traditional medicine. J. Ethnopharmacol..

[68] Shahat A.A., Mahmoud E.A., Al-Mishari A.A., Alsaid M.S. (2017). Antimicrobial activities of some Saudi Arabian herbal plants. Afr. J. Trad. Complement. Altern. Med..

[69] Cioch M., Satora P., Skotniczny M., Semik-Szczurak D., Tarko T. (2017). Characterisation of Antimicrobial Properties of Extracts of Selected Medicinal Plants. Pol. J. Microbiol..

[70] Voukeng I.K., Beng V.P., Kuete V. (2016). Antibacterial activity of six medicinal Cameroonian plants against Gram-positive and Gram-negative multidrug resistant phenotypes. BMC Complement. Altern. Med..

[71] Anyanwu M.U., Okoye R.C. (2017). Antimicrobial activity of Nigerian medicinal plants. J. Intercult Ethnopharmacol..

[72] Othman L., Sleiman A., Abdel-Massih R.M. (2019). Antimicrobial Activity of Polyphenols and Alkaloids in Middle Eastern Plants. Front. Microbiol..

[73] Ganesan K., Xu B. (2017). Telomerase Inhibitors from Natural Products and Their Anticancer Potential. Int. J. Mol. Sci..

[74] Sukalingam K., Ganesan K., Xu B. (2018). Protective Effect of Aqueous Extract from the Leaves of Justicia tranquebariesis against Thioacetamide-Induced Oxidative Stress and Hepatic Fibrosis in Rats. Antioxidants.

[75] Ganesan K., Xu B. (2017). Polyphenol-Rich Lentils and Their Health Promoting Effects. Int. J.Mol. Sci..

[76] Ganesan K., Xu B. (2017). Molecular targets of vitexin and isovitexin in cancer therapy: A critical review. Ann. N. Y. Acad. Sci..

[77] Ganesan K., Gani S.B., Ganesan Murugesan A. (2008). Influence of Helicteres isora L. bark extracts on glycemic control and renoprotective activity in streptozotocin-induced diabetic rats. Int. J. Pharm. Sci. Nanotechnol..

[78] Kumar G., Murugesan A.G. (2008). Hypolipidaemic activity of Helicteres isora L. bark extracts in streptozotocin induced diabetic rats. J. Ethnopharmacol..

[79] Kumar G., Banu G.S., Murugesan A.G., Pandian M.R. (2006). Hypoglycaemic effect of Helicteres isora bark extract in rats. J. Ethnopharmacol..

[80] Kumar G., Sharmila Banu G., Murugesan A.G., Rajasekara Pandian M. (2007). Effect ofHelicteres isora. Bark Extracts on Brain Antioxidant Status and Lipid Peroxidation in Streptozotocin Diabetic Rats. Pharm. Biol..

[81] Kumar G., Sharmila Banu G., Ganesan Murugesan A. (2008). Effect of Helicteres isora bark extracts on heart antioxidant status and lipid peroxidation in streptozotocin diabetic rats. J. Appl. Biomed..

[82] Ganesan K., Xu B. (2018). Anti-Obesity Effects of Medicinal and Edible Mushrooms. Molecules.

[83] Ganesan K., Gani S.B., Ganesan Murugesan A. (2009). Antidiabetic activity of Helicteres isora L. bark extracts on streptozotocin-induced diabetic rats. Int. J. Pharm. Sci. Nanotechnol..

[84] Ganesan K., Chung S.K., Vanamala J., Xu B. (2018). Causal Relationship between Diet-Induced Gut Microbiota Changes and Diabetes: A Novel Strategy to Transplant Faecalibacterium prausnitzii in Preventing Diabetes. Int. J. Mol. Sci..

[85] Górniak I., Bartoszewski R., Króliczewski J. (2019). Comprehensive review of antimicrobial activities of plant flavonoids. Phytochem. Rev..

[86] Madikizela B., Aderogba M.A., Van Staden J. (2013). Isolation and characterization of antimicrobial constituents of Searsia chirindensis L. (Anacardiaceae) leaf extracts. J. Ethnopharmacol..

[87] Alolga R.N., Chávez León M.A.S.C., Osei-Adjei G., Onoja V. (2019). GC-MS-based metabolomics, antibacterial and anti-inflammatory investigations to characterize the quality of essential oil obtained from dried Xylopia aethiopica fruits from Ghana and Nigeria. J. Pharm. Pharmacol..

[88] Vieira M.L.A., Johann S., Hughes F.M., Rosa C.A., Rosa L.H. (2014). The diversity and antimicrobial activity of endophytic fungi associated with medicinal plant Baccharis trimera (Asteraceae) from the Brazilian savannah. Can. J. Microbiol..

[89] Lourenção Brighenti F., Salvador M.J., Vidal Lacerda Gontijo A., Botazzo Delbem A.C., Botazzo Delbem Á.C., Soares C.P., Carvalho de Oliveira M.A., Miorelli Girondi C., Koga-Ito C.Y. (2017). Plant extracts: Initial screening, identification of bioactive compounds and effect against Candida albicansbiofilms. Fut. Microbiol..

[90] Abu-Darwish M.S., Cabral C., Gonçalves M.J., Cavaleiro C., Cruz M.T., Efferth T., Salgueiro L. (2015). Artemisia herba-alba essential oil from Buseirah (South Jordan): Chemical characterization and assessment of safe antifungal and anti-inflammatory doses. J. Ethnopharmacol..

[91] Gazoni V.F., Balogun S.O., Arunachalam K., Oliveira D.M., Filho V.C., Lima S.R., Colodel E.M., Soares I.M., Ascêncio S.D., Martins D.T.d.O. (2018). Assessment of toxicity and differential antimicrobial activity of methanol extract of rhizome of Simaba ferruginea A. St.-Hil. and its isolate canthin-6-one. J. Ethnopharmacol..

[92] Perianayagam J.B., Sharma S.K., Pillai K.K., Pandurangan A., Kesavan D. (2012). Evaluation of antimicrobial activity of ethanol extract and compounds isolated from Trichodesma indicum (Linn.) R. Br. root. J. Ethnopharmacol..

[93] Dandashire B., Magashi A., Abdulkadir B., Abbas M., Goni M., Yakubu A. (2019). Toxicological studies and bioactivity-guided identification of antimicrobially active compounds from crude aqueous stem bark extract of Boswellia dalzielii. J. Advan. Vet. Anim. Res..

[94] Olmedo-Juárez A., Briones-Robles T.I., Zaragoza-Bastida A., Zamilpa A., Ojeda-Ramírez D., Mendoza de Gives P., Olivares-Pérez J., Rivero-Perez N. (2019). Antibacterial activity of compounds isolated from Caesalpinia coriaria (Jacq) Willd against important bacteria in public health. Microb. Pathog..

[95] Madikizela B., Aderogba M.A., Finnie J.F., Van Staden J. (2014). Isolation and characterization of antimicrobial compounds from Terminalia phanerophlebia Engl. & Diels leaf extracts. J. Ethnopharmacol..

[96] Teodoro G.R., Brighenti F.L., Delbem A.C.B., Delbem Á.C.B., Khouri S., Gontijo A.V.L., Pascoal A.C.R.F., Salvador M.J., Koga-Ito C.Y. (2015). Antifungal activity of extracts and isolated compounds fromBuchenavia tomentosaonCandida albicansand non-albicans. Fut. Microbiol..

[97] Pramila D.M. (2012). Phytochemical analysis and antimicrobial potential of methanolic leaf extract of peppermint (Mentha piperita: Lamiaceae). J. Med. Plants Res..

[98] Rezzoug M., Bakchiche B., Gherib A., Roberta A., FlaminiGuido, Kilinçarslan Ö., Mammadov R., Bardaweel S.K. (2019). Chemical composition and bioactivity of essential oils and Ethanolic extracts of Ocimum basilicum L. and Thymus algeriensis Boiss. & Reut. from the Algerian Saharan Atlas. BMC Complement. Altern. Med..

[99] Mustaffa F., Indurkar J., Ismail S., Shah M., Mansor S.M. (2011). An Antimicrobial Compound Isolated from Cinnamomum Iners Leaves with Activity against Methicillin-Resistant Staphylococcus Aureus. Molecules.

[100] Xu X., Zhou X.D., Wu C.D. (2010). The Tea Catechin Epigallocatechin Gallate Suppresses Cariogenic Virulence Factors ofStreptococcus mutans. Antimicrob. Agents Chemother..

[101] Kuete V., Ango P.Y., Fotso G.W., Kapche G.D.W.F., Dzoyem J.P., Wouking A.G., Ngadjui B.T., Abegaz B.M. (2011). Antimicrobial activities of the methanol extract and compounds from Artocarpus communis (Moraceae). BMC Complement. Altern. Med..

[102] Bouzabata A., Bazzali O., Cabral C., Gonçalves M.J., Cruz M.T., Bighelli A., Cavaleiro C., Casanova J., Salgueiro L., Tomi F. (2013). New compounds, chemical composition, antifungal activity and cytotoxicity of the essential oil from Myrtus nivellei Batt. & Trab., an endemic species of Central Sahara. J. Ethnopharmacol..

[103] González-Alamilla E.N., Gonzalez-Cortazar M., Valladares-Carranza B., Rivas-Jacobo M.A., Herrera-Corredor C.A., Ojeda-Ramírez D., Zaragoza-Bastida A., Rivero-Perez N. (2019). Chemical Constituents of Salix babylonica L. and Their Antibacterial Activity Against Gram-Positive and Gram-Negative Animal Bacteria. Molecules.

[104] Xu X., Zhou X.D., Wu C.D. (2012). Tea catechin epigallocatechin gallate inhibits Streptococcus mutans biofilm formation by suppressing gtf genes. Arch. Oral Biol..

[105] Ganesan K., Xu B. (2017). Polyphenol-Rich Dry Common Beans (Phaseolus vulgaris L.) and Their Health Benefits. Int. J. Mol. Sci..

[106] Stapleton P. (2004). Anti-Staphylococcus aureus activity and oxacillin resistance modulating capacity of 3-O-acyl-catechins. Int. J. Antimicrob. Agents.

[107] Cushnie T.P.T., Hamilton V.E.S., Chapman D.G., Taylor P.W., Lamb A.J. (2007). Aggregation of Staphylococcus aureus following treatment with the antibacterial flavonol galangin. J. Appl. Microbiol..

[108] El-Adawi H. (2012). Inhibitory effect of grape seed extract (GSE) on cariogenic bacteria. J. Med. Plants Res..

[109] Awolola G.V., Koorbanally N.A., Chenia H., Shode F.O., Baijnath H. (2014). Antibacterial and Anti-Biofilm Activity of Flavonoids and Triterpenes Isolated from The Extracts of Ficus Sansibarica Warb. Subsp. Sansibarica (Moraceae) Extracts. Afr. J. Trad. Complement. Altern. Med..

[110] Ganesan K., Xu B. (2018). A critical review on phytochemical profile and health promoting effects of mung bean (Vigna radiata ). Food Sci. Hum. Wellness.

[111] Ganesan K., Xu B. (2017). A Critical Review on Polyphenols and Health Benefits of Black Soybeans. Nutrients.

[112] Tsuchiya H., Iinuma M. (2000). Reduction of membrane fluidity by antibacterial sophoraflavanone G isolated from Sophora exigua. Phytomedicine.

[113] Sanver D., Murray B.S., Sadeghpour A., Rappolt M., Nelson A.L. (2016). Experimental Modeling of Flavonoid–Biomembrane Interactions. Langmuir.

[114] Stepanović S., Antić N., Dakić I., Švabić-Vlahović M. (2003). In vitro antimicrobial activity of propolis and synergism between propolis and antimicrobial drugs. Microbiol. Res..

[115] Ollila F., Halling K., Vuorela P., Vuorela H., Slotte J.P. (2002). Characterization of Flavonoid–Biomembrane Interactions. Arch. Biochem. Biophys..

[116] Mishra A.K., Mishra A., Kehri H.K., Sharma B., Pandey A.K. (2009). Inhibitory activity of Indian spice plant Cinnamomum zeylanicum extracts against Alternaria solani and Curvularia lunata, the pathogenic dematiaceous moulds. Ann. Clin. Microbiol. Antimicrob..

[117] Matijašević D., Pantić M., Rašković B., Pavlović V., Duvnjak D., Sknepnek A., Nikšić M. (2016). The Antibacterial Activity of Coriolus versicolor Methanol Extract and Its Effect on Ultrastructural Changes of Staphylococcus aureus and Salmonella Enteritidis. Front. Microbiol..

[118] Budzynska A., Rozalski M., Karolczak W., Wieckowska-Szakiel M., Sadowska B., Rozalska B. (2011). Synthetic 3-Arylidenefl avanones as Inhibitors of the Initial Stages of Biofilm Formation by Staphylococcus aureus and Enterococcus faecalis. Z. Für Naturforschung C.

[119] Ganesan K., Jayachandran M., Xu B. (2017). A critical review on hepatoprotective effects of bioactive food components. Crit. Rev. Food Sci. Nutr..

[120] Srikrishna D., Godugu C., Dubey P.K. (2018). A Review on Pharmacological Properties of Coumarins. Mini-Rev. Med. Chem..

[121] Guimarães A.C., Meireles L.M., Lemos M.F., Guimarães M.C.C., Endringer D.C., Fronza M., Scherer R. (2019). Antibacterial Activity of Terpenes and Terpenoids Present in Essential Oils. Molecules.

[122] Moghrovyan A., Sahakyan N., Babayan A., Chichoyan N., Petrosyan M., Trchounian A. (2019). Essential Oil and Ethanol Extract of Oregano (Origanum vulgare L.) from Armenian Flora as a Natural Source of Terpenes, Flavonoids and other Phytochemicals with Antiradical, Antioxidant, Metal Chelating, Tyrosinase Inhibitory and Antibacterial Activity. Curr. Pharm. Des..

[123] Lambert R.J.W., Skandamis P.N., Coote P.J., Nychas G.J.E. (2001). A study of the minimum inhibitory concentration and mode of action of oregano essential oil, thymol and carvacrol. J. Appl. Microbiol..

[124] Ultee A., Bennik M.H.J., Moezelaar R. (2002). The Phenolic Hydroxyl Group of Carvacrol Is Essential for Action against the Food-Borne Pathogen Bacillus cereus. Appl. Environ. Microbiol..

[125] Silva N.C.C., Fernandes Júnior A. (2010). Biological properties of medicinal plants: A review of their antimicrobial activity. J. Venom. Anim. Toxins Trop. Dis..

[126] Vikram A., Jayaprakasha G.K., Jesudhasan P.R., Pillai S.D., Patil B.S. (2010). Suppression of bacterial cell-cell signalling, biofilm formation and type III secretion system by citrus flavonoids. J. Appl. Microbiol..

[127] Prasad V.G.N.V., krishna B.V., Swamy P.L., Rao T.S., Rao G.S. (2014). Antibacterial synergy between quercetin and polyphenolic acids against bacterial pathogens of fish. Asian Pac. J. Trop. Dis..

[128] Thiago J.D.S.B., Andréa F.F., Ana C.D.P.R.I., Norma A. (2016). Cytotoxic, antibacterial and antibiofilm activities of aqueous extracts of leaves and flavonoids occurring in Kalanchoe pinnata (Lam.) Pers. J. Med. Plants Res..

[129] Ouyang J., Sun F., Feng W., Sun Y., Qiu X., Xiong L., Liu Y., Chen Y. (2016). Quercetin is an effective inhibitor of quorum sensing, biofilm formation and virulence factors inPseudomonas aeruginosa. J. Appl. Microbiol..

[130] Ulrey R.K., Barksdale S.M., Zhou W., van Hoek M.L. (2014). Cranberry proanthocyanidins have anti-biofilm properties against Pseudomonas aeruginosa. BMC Complement. Altern. Med..

[131] Paczkowski J.E., Mukherjee S., McCready A.R., Cong J.-P., Aquino C.J., Kim H., Henke B.R., Smith C.D., Bassler B.L. (2017). Flavonoids SuppressPseudomonas aeruginosaVirulence through Allosteric Inhibition of Quorum-sensing Receptors. J. Biol. Chem..

[132] Roy R., Tiwari M., Donelli G., Tiwari V. (2017). Strategies for combating bacterial biofilms: A focus on anti-biofilm agents and their mechanisms of action. Virulence.

[133] Oteiza P.I., Erlejman A.G., Verstraeten S.V., Keen C.L., Fraga C.G. (2005). Flavonoid-membrane Interactions: A Protective Role of Flavonoids at the Membrane Surface?. Clin. Dev. Immunol..

[134] Vasconcelos M.A., Arruda F.V.S., de Alencar D.B., Saker-Sampaio S., Albuquerque M.R.J.R., dos Santos H.S., Bandeira P.N., Pessoa O.D.L., Cavada B.S., Henriques M. (2014). Antibacterial and Antioxidant Activities of Derriobtusone A Isolated from Lonchocarpus obtusus. Biomed. Res. Int..

[135] Christena L.R., Subramaniam S., Vidhyalakshmi M., Mahadevan V., Sivasubramanian A., Nagarajan S. (2015). Dual role of pinostrobin-a flavonoid nutraceutical as an efflux pump inhibitor and antibiofilm agent to mitigate food borne pathogens. RSC Adv..

[136] Lee P., Tan K.S. (2015). Effects of Epigallocatechin gallate against Enterococcus faecalis biofilm and virulence. Arch. Oral Biol..

[137] Reygaert W.C. (2014). The antimicrobial possibilities of green tea. Front. Microbiol..

[138] Zhang L., Kong Y., Wu D., Zhang H., Wu J., Chen J., Ding J., Hu L., Jiang H., Shen X. (2008). Three flavonoids targeting the β-hydroxyacyl-acyl carrier protein dehydratase from Helicobacter pylori: Crystal structure characterization with enzymatic inhibition assay. Protein Sci..

[139] Jeong K.-W., Lee J.-Y., Kang D.-I., Lee J.-U., Shin S.Y., Kim Y. (2009). Screening of Flavonoids as Candidate Antibiotics againstEnterococcus faecalis. J. Nat. Prod..

[140] Zhang Y.-M., Rock C.O. (2004). Evaluation of Epigallocatechin Gallate and Related Plant Polyphenols as Inhibitors of the FabG and FabI Reductases of Bacterial Type II Fatty-acid Synthase. J. Biol. Chem..

[141] Elmasri W.A., Zhu R., Peng W., Al-Hariri M., Kobeissy F., Tran P., Hamood A.N., Hegazy M.F., Paré P.W., Mechref Y. (2017). Multitargeted Flavonoid Inhibition of the Pathogenic Bacterium *Staphylococcus aureus*: A Proteomic Characterization. J. Proteome Res..

[142] Zhang F., Luo S.-Y., Ye Y.-B., Zhao W.-H., Sun X.-G., Wang Z.-Q., Li R., Sun Y.-H., Tian W.-X., Zhang Y.-X. (2008). The antibacterial efficacy of an aceraceous plant [Shantung maple (Acer truncatum Bunge)] may be related to inhibition of bacterial β-oxoacyl-acyl carrier protein reductase (FabG). Biotechnol. Appl. Biochem..

[143] Brown A.K., Papaemmanouil A., Bhowruth V., Bhatt A., Dover L.G., Besra G.S. (2007). Flavonoid inhibitors as novel antimycobacterial agents targeting Rv0636, a putative dehydratase enzyme involved in Mycobacterium tuberculosis fatty acid synthase II. Microbiology.

[144] Fujita M., Shiota S., Kuroda T., Hatano T., Yoshida T., Mizushima T., Tsuchiya T. (2005). Remarkable Synergies between Baicalein and Tetracycline, and Baicalein and β-Lactams against Methicillin-ResistantStaphylococcus aureus. Microbiol. Immunol..

[145] Eumkeb G., Siriwong S., Phitaktim S., Rojtinnakorn N., Sakdarat S. (2011). Synergistic activity and mode of action of flavonoids isolated from smaller galangal and amoxicillin combinations against amoxicillin-resistant Escherichia coli. J. Appl. Microbiol..

[146] Vijayakumar R., Sandle T., Al-Aboody M.S., AlFonaisan M.K., Alturaiki W., Mickymaray S., Premanathan M., Alsagaby S.A. (2018). Distribution of biocide resistant genes and biocides susceptibility in multidrug-resistant Klebsiella pneumoniae, Pseudomonas aeruginosa and Acinetobacter baumannii - A first report from the Kingdom of Saudi Arabia. J. Infect. Public Health.

[147] Zielińska S., Wójciak-Kosior M., Dziągwa-Becker M., Gleńsk M., Sowa I., Fijałkowski K., Rurańska-Smutnicka D., Matkowski A., Junka A. (2019). The Activity of Isoquinoline Alkaloids and Extracts from Chelidonium majus against Pathogenic Bacteria and Candida sp.. Toxins.

[148] Plaper A., Golob M., Hafner I., Oblak M., Šolmajer T., Jerala R. (2003). Characterization of quercetin binding site on DNA gyrase. Biochem. Biophy. Res. Comm..

[149] Verdrengh M., Collins L.V., Bergin P., Tarkowski A. (2004). Phytoestrogen genistein as an anti-staphylococcal agent. Microb. Infect..

[150] Ulanowska K., Tkaczyk A., Konopa G., Węgrzyn G. (2005). Differential antibacterial activity of genistein arising from global inhibition of DNA, RNA and protein synthesis in some bacterial strains. Arch. Microbiol..

[151] Wu D., Kong Y., Han C., Chen J., Hu L., Jiang H., Shen X. (2008). d-Alanine:d-alanine ligase as a new target for the flavonoids quercetin and apigenin. Int. J. Antimicrob. Agents.

[152] Xu H. (2001). Flavones inhibit the hexameric replicative helicase RepA. Nucleic Acids Res..

[153] Shadrick W.R., Ndjomou J., Kolli R., Mukherjee S., Hanson A.M., Frick D.N. (2013). Discovering New Medicines Targeting Helicases. J. Biomol. Screen..

[154] Bhosle A., Chandra N. (2016). Structural analysis of dihydrofolate reductases enables rationalization of antifolate binding affinities and suggests repurposing possibilities. FEBS J..

[155] Navarro-Martinez M.D., Navarro-Peran E., Cabezas-Herrera J., Ruiz-Gomez J., Garcia-Canovas F., Rodriguez-Lopez J.N. (2005). Antifolate Activity of Epigallocatechin Gallate against Stenotrophomonas maltophilia. Antimicrob. Agents Chemother..

[156] Raju A., Degani M.S., Khambete M.P., Ray M.K., Rajan M.G.R. (2015). Antifolate Activity of Plant Polyphenols againstMycobacterium tuberculosis. Phytother. Res..

[157] Walker E.H., Pacold M.E., Perisic O., Stephens L., Hawkins P.T., Wymann M.P., Williams R.L. (2000). Structure determinants of phosphoinositide 3-kinase inhibition by wortmannin, LY294002, quercetin, myricetin and staurosporine. Mol. Cell.

[158] Gledhill J.R., Montgomery M.G., Leslie A.G.W., Walker J.E. (2007). Mechanism of inhibition of bovine F1-ATPase by resveratrol and related polyphenols. Proc. Natl. Acad. Sci. USA.

[159] Chinnam N., Dadi P.K., Sabri S.A., Ahmad M., Kabir M.A., Ahmad Z. (2010). Dietary bioflavonoids inhibit Escherichia coli ATP synthase in a differential manner. Int. J. Biol. Macromol..

[160] Shah S., Stapleton P.D., Taylor P.W. (2007). The polyphenol (−)-epicatechin gallate disrupts the secretion of virulence-related proteins by Staphylococcus aureus. Lett. Appl. Microbiol..

[161] Lee J.-H., Regmi S.C., Kim J.-A., Cho M.H., Yun H., Lee C.-S., Lee J. (2011). Apple Flavonoid Phloretin Inhibits Escherichia coli O157:H7 Biofilm Formation and Ameliorates Colon Inflammation in Rats. Infect. Immun..

[162] Choi O., Yahiro K., Morinaga N., Miyazaki M., Noda M. (2007). Inhibitory effects of various plant polyphenols on the toxicity of Staphylococcal α-toxin. Microb. Pathog..

[163] Ruddock P.S., Charland M., Ramirez S., López A., Neil Towers G.H., Arnason J.T., Liao M., Dillon J.-A.R. (2011). Antimicrobial Activity of Flavonoids From Piper lanceaefolium and Other Colombian Medicinal Plants Against Antibiotic Susceptible and Resistant Strains of Neisseria gonorrhoeae. Sex. Transm. Dis..

[164] Rasul A., Millimouno F.M., Ali Eltayb W., Ali M., Li J., Li X. (2013). Pinocembrin: A Novel Natural Compound with Versatile Pharmacological and Biological Activities. Biomed Res. Int..

[165] Ahmed S.I., Hayat M.Q., Tahir M., Mansoor Q., Ismail M., Keck K., Bates R.B. (2016). Pharmacologically active flavonoids from the anticancer, antioxidant and antimicrobial extracts of Cassia angustifolia Vahl. Bmc Complement. Altern. Med..

[166] Girish K.S., Kemparaju K. (2007). The magic glue hyaluronan and its eraser hyaluronidase: A biological overview. Life Sci..

[167] Hertel W., Peschel G., Ozegowski J.-H., Müller P.-J. (2006). Inhibitory Effects of Triterpenes and Flavonoids on the Enzymatic Activity of Hyaluronic Acid-Splitting Enzymes. Arch. Pharm..

[168] Bush K., Fisher J.F. (2011). Epidemiological Expansion, Structural Studies, and Clinical Challenges of New β-Lactamases from Gram-Negative Bacteria. Annu. Rev. Microbiol..

[169] Bush K. (2013). The ABCD’s of β-lactamase nomenclature. J. Infect. Chemother..

[170] Munita J.M., Arias C.A. (2016). Mechanisms of Antibiotic Resistance. Virulence Mechanisms of Bacterial Pathogens.

[171] Yan X., Gu S., Shi Y., Cui X., Wen S., Ge J. (2017). The effect of emodin on Staphylococcus aureus strains in planktonic form and biofilm formation in vitro. Arch. Microbiol..

[172] Peng Q., Zhou S., Yao F., Hou B., Huang Y., Hua D., Zheng Y., Qian Y. (2011). Baicalein Suppresses the SOS Response System of Staphylococcus Aureus Induced by Ciprofloxacin. Cell. Physiol. Biochem..

[173] Klančnik A., Možina S.S., Zhang Q. (2012). Anti-Campylobacter Activities and Resistance Mechanisms of Natural Phenolic Compounds in Campylobacter. PLoS ONE.

[174] Fathima A., Rao J.R. (2016). Selective toxicity of Catechin—A natural flavonoid towards bacteria. Appl. Microbiol. Biotechnol..

[175] Cushnie T.P.T., Taylor P.W., Nagaoka Y., Uesato S., Hara Y., Lamb A.J. (2008). Investigation of the antibacterial activity of 3-O-octanoyl-(-)-epicatechin. J. Appl. Microbiol..

